# Recruitment of BAF to the nuclear envelope couples the LINC complex to endoreplication

**DOI:** 10.1242/dev.191304

**Published:** 2020-12-13

**Authors:** C. P. Unnikannan, Adriana Reuveny, Dvorah Grunberg, Talila Volk

**Affiliations:** Department of Molecular Genetics, Weizmann Institute of Science, Rehovot 76100, Israel

**Keywords:** BAF, Endoreplication, LINC complex, Nuclear lamina, Muscle, Nuclear envelope

## Abstract

DNA endoreplication has been implicated as a cell strategy for cell growth and in tissue injury. Here, we demonstrate that barrier-to-autointegration factor (BAF) represses endoreplication in *Drosophila* myofibers. We show that BAF localization at the nuclear envelope is eliminated in flies with mutations of the linker of nucleoskeleton and cytoskeleton (LINC) complex in which the LEM-domain protein Otefin is excluded, or after disruption of the nucleus-sarcomere connections. Furthermore, BAF localization at the nuclear envelope requires the activity of the BAF kinase VRK1/Ball, and, consistently, non-phosphorylatable BAF-GFP is excluded from the nuclear envelope. Importantly, removal of BAF from the nuclear envelope correlates with increased DNA content in the myonuclei. E2F1, a key regulator of endoreplication, overlaps BAF localization at the myonuclear envelope, and BAF removal from the nuclear envelope results in increased E2F1 levels in the nucleoplasm and subsequent elevated DNA content. We suggest that LINC-dependent and phosphosensitive attachment of BAF to the nuclear envelope, through its binding to Otefin, tethers E2F1 to the nuclear envelope thus inhibiting its accumulation in the nucleoplasm.

## Abstract (precis)

**Summary:** Localization of BAF at the nuclear envelope of myonuclei depends on a functional LINC complex and on nucleus-sarcomere connections, and is shown to restrict E2F1 levels in the nucleoplasm.

## INTRODUCTION

Endoreplication emerges as an important strategy of differentiated cells, enabling them to grow in size or rescue tissue integrity following injury, in a wide range of non-dividing cell types (Gan et al., 2019). Recent experimental studies have proposed a functional link between mechanical inputs and endoreplication events in various cell types (Cao et al., 2017; Sun et al., 2019). Moreover, mechanical signals transmitted across the nuclear membrane have been implicated in the regulation of cell cycle, epigenetic events and gene transcription (Cao et al., 2017; Cho et al., 2017; Kirby and Lammerding, 2018). As part of the mechanism linking cell cycle events with mechanical inputs, the translocation of specific essential factors into the nucleus has been proposed (Aragona et al., 2013; Driscoll et al., 2015; Dupont et al., 2011; Ho et al., 2013; Kassianidou et al., 2019). However, the molecular link between nuclear translocation of such factors, mechanical inputs on the nuclear envelope and endoreplication is still elusive.

The linker of nucleoskeleton and cytoskeleton (LINC) complex has been suggested to mediate mechanically induced nuclear entry of essential factors (Driscoll et al., 2015; Horn, 2014; Osmanagic-Myers et al., 2015). It physically connects the cytoskeleton and the nucleoskeleton at the interface of the nuclear envelope and has been associated with various human myopathies (Horn, 2014; Mejat and Misteli, 2010; Mellad et al., 2010). The LINC complex is composed of Nesprin protein family members, which associate at their cytoplasmic N-terminal end with distinct cytoskeletal components, and on their nuclear C-terminal end with SUN domain proteins at the perinuclear space (Rajgor and Shanahan, 2013; Zhang et al., 2002, 2001). SUN domain proteins bind to various nuclear lamina components, resulting in a physical link between the cytoskeleton and the nucleoskeleton (Cain and Starr, 2015; Lombardi et al., 2011; Padmakumar et al., 2005; Sosa et al., 2012). Recent results from our laboratory indicate that, in *Drosophila* larval muscles, the LINC complex is essential for arresting endoreplication in the muscle nuclei (myonuclei) and that LINC mutants exhibit additional rounds of DNA replication, resulting in elevated polyploidy (Brayson et al., 2018; Volk, 2012; Wang et al., 2018). The molecular nature of this process is currently elusive.

In an attempt to reveal the components downstream of the LINC-dependent arrest of DNA endoreplication, we performed a screen for genes whose transcription changes in *Drosophila* Nesprin/*klar* mutant muscles. One of the identified genes was *barrier-to-autointegration factor* (*baf*), shown to be significantly reduced at the transcription level (Wang et al., 2018). BAF is a small protein of 89 amino acids that binds dsDNA as well as the nuclear envelope, and in addition forms homodimers (Bradley et al., 2005; Zheng et al., 2000). Furthermore, BAF binds to the inner components of the nuclear membrane, including the Lap-2, Emerin, MAN1 (LEM) domain proteins, as well as to lamins A/C and B. Thus, BAF dimers might bridge between dsDNA and the nuclear envelope (Cai et al., 2001; Lee et al., 2001; Mansharamani and Wilson, 2005; Samwer et al., 2017). Proteomic analysis of BAF partners indicate its potential association with additional proteins, including transcription factors, damage-specific DNA binding proteins and histones (Holaska et al., 2003; Montes de Oca et al., 2009). Furthermore, the binding of BAF to its potential partners might be regulated by its phosphorylation state (Birendra et al., 2017; Lancaster et al., 2007; Nichols et al., 2006). For example, phosphorylated BAF associates with LEM-domain proteins, whereas de-phosphorylated BAF favors binding to dsDNA (Bengtsson and Wilson, 2006; Nichols et al., 2006). One kinase that has been implicated in BAF phosphorylation is the threonine-serine VRK1 kinase, whose homolog in *Drosophila* is Ballchen (Ball, also known as NHK-1) (Lancaster et al., 2007).

BAF has a crucial role in the condensation and assembly of post-mitotic DNA. Its interaction with both dsDNA and the nuclear lamina enables DNA compaction through cross-bridges between chromosomes and the nuclear envelope, a process essential for the assembly of DNA within a single nucleus following mitosis (Samwer et al., 2017). Likewise, BAF is recruited to the sites of ruptured nuclear membrane, where it is essential for resealing the ruptured nuclear membrane (Halfmann et al., 2019). Interestingly, in humans a single amino acid substitution of BAF causes Nestor–Guillermo progeria syndrome (NGPS) (Puente et al., 2011); however, the molecular basis for the disease awaits further investigation.

Previously, we demonstrated that in *Drosophila*, muscle-specific knockdown of BAF increases the levels of DNA endoreplication, phenocopying the LINC mutant outcome (Wang et al., 2018). This led to the hypothesis that BAF acts downstream of the LINC complex-dependent mechanotransduction in promoting the arrest of DNA endoreplication in muscle. Here, we demonstrate that BAF localization at the nuclear envelope is crucial for that process, and that it is downstream of the LINC complex, depends on nucleus-sarcomere connections, and is phosphosensitive. Importantly, elimination of BAF from the nuclear envelope correlates with increased DNA content in the myonuclei and a concomitant increase in E2F1 levels in the nucleoplasm. Taken together, our findings suggest a model in which a LINC-dependent localization of BAF at the nuclear envelope promotes E2F1 tethering to the nuclear envelope to inhibit its accumulation in the nucleoplasm.

## RESULTS

### BAF localization at the nuclear envelope depends on a functional LINC complex

**Fig. 1.**
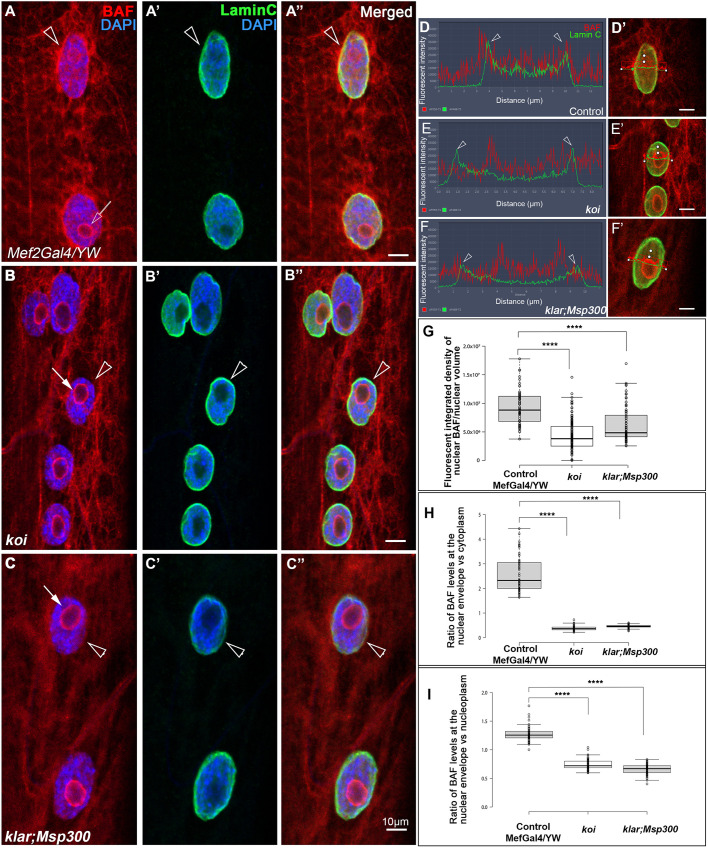
**BAF dissociates from the nuclear envelope in the LINC mutant muscles.** (A-C″) Representative larval muscle no. 7 of control (*Mef2Gal4*/YW; A-A″), LINC mutant *klaroid/SUN* (*koi*) (B-B″) or LINC double mutant combination of *klar^ΔKASH^* and Msp^ΔKASH^ (*klar;Msp300*; C-C″). Muscles are labeled with anti-BAF and DAPI (red, blue; A,B,C) or with anti-Lamin C and DAPI (green, blue; A′,B′,C′). Merged images are shown in A″, B″,C″. Arrows indicate the nucleolus and arrowheads indicate the nuclear membrane. (D-F′) Representative line profiles of BAF (red) and anti-Lamin C (green), taken at the middle of each myonucleus of control (D), *koi* mutants (E) or *klar;Msp300* mutants (F) and their corresponding nuclei (D′,E′,F′). Arrowheads in D,E,F indicate the lamin peaks at the nuclear envelope borders. All images represent single confocal Z stacks. (G) Quantification of the fluorescence integrated density of BAF per nucleus in control, *koi* and *klar;Msp300* mutant myonuclei. (H) The ratio between BAF fluorescence at the nuclear envelope and BAF fluorescence in the cytoplasm. (I) The ratio between BAF fluorescence at the nuclear envelope and BAF fluorescence in the nucleoplasm. Each of the experiments was repeated 3 times, indicating similar trends. Quantifications were calculated from *n*=66 myonuclei of control, *n*=136 myonuclei of *koi* mutants and *n*=74 myonuclei of *klar;Msp300* mutants. One-tailed *t*-test for each of the LINC mutant groups relative to control groups indicates a significant difference between control and mutant groups (****P<0.0001). Scale bars: 10 µm.

Previously we showed that the transcription of BAF is significantly reduced in *Drosophila* larval muscles in the LINC mutants *klar* and *koi*, and in addition, knockdown of BAF in these muscles leads to elevated DNA content in the myonuclei (Wang et al., 2018). We used anti-BAF antibodies (Furukawa et al., 2003) to reveal the cellular distribution of BAF protein in the LINC mutant larval muscles. In control myonuclei, BAF accumulated at the nuclear envelope, overlapping Lamin C (Fig. 1A-A″,D,D′), and at the nucleolus borders as well as partially overlapping the microtubules (Fig. 1A; Fig. S1A,B). Importantly, BAF labeling at the nuclear envelope was specifically eliminated in the LINC mutant *klaroid* (*koi*)*,* lacking a SUN domain protein, and in double homozygous mutants of *klar* and *Msp30*0 alleles (*klar;Msp300*), lacking only the Klarsicht, ANC-1, syne homology (KASH) domain (Fig. 1B-C″,E,E′,F,F′). Together, these mutants represent the entire repertoire of *Drosophila* LINC complex genes. BAF localization at the nucleolus borders did not change in the LINC mutants (Fig. 1B-C″,E,F). Quantification of BAF-positive fluorescent voxels within the entire nuclear volume defined by Lamin C borders (see Materials and Methods), normalized to nuclear volume, indicated a statistically significant reduction in the fluorescence levels of nuclear BAF in both *koi* and *klar;Msp300* LINC mutants (Fig. 1G). In addition, we calculated the ratio between BAF fluorescence at the nuclear envelope relative to its cytoplasmic levels or, alternatively, to its levels in the nucleoplasm (Fig. 1H,I). In both cases, a significant decrease in the relative localization of BAF at the nuclear envelope was observed. The difference between each of the mutant groups relative to control was statistically significant (*P*<0.0001). Taken together, these results imply that the specific localization of BAF protein at the nuclear envelope requires a functional LINC complex.

**Fig. 2.**
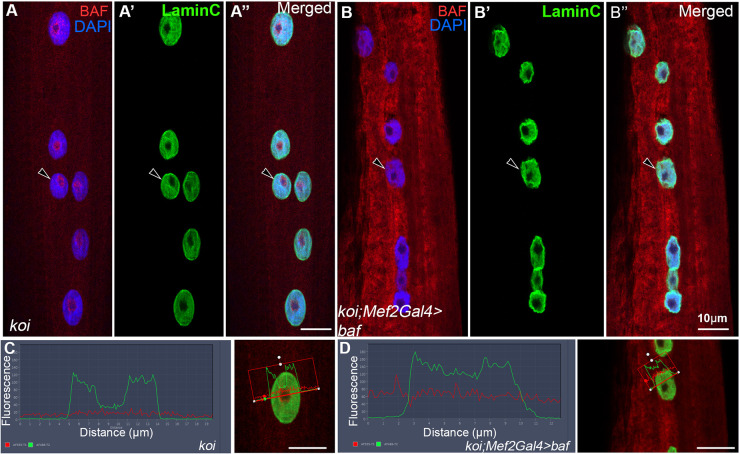
**Overexpression of BAF in *koi* mutant muscles does not rescue BAF localization at the nuclear envelope.** (A-B″) Representative larval muscle no. 7 of homozygous *koi* mutant (A-A″) or *koi* mutant overexpressing BAF in muscles under the control of *Mef2Gal4* driver (B-B″), labeled with anti-BAF and DAPI (red and blue) or anti-Lamin C (green). Merged images are shown in A″,B″. Arrowheads indicate the nuclear envelope border. (C,D) Line profiles of a single nucleus of *koi* mutant (C; the nucleus analyzed is indicated by arrowheads in A-A″ and is shown in the right panel of C) or *koi* mutant overexpressing BAF (D; the nucleus analyzed is indicated by arrowheads in B-B″ and is shown in the right panel of D). All images represent a single confocal Z stack. Scale bars: 10 µm.

Previously we showed that BAF transcription is reduced in *koi* and *klar* LINC mutants (Wang et al., 2018). To exclude a possible effect of BAF reduction on its localization at the nuclear envelope, we attempted to overexpress BAF in muscles of *koi* mutants. However, despite a general increase in its cytoplasmic levels, BAF overexpression in *koi* mutant muscles did not rescue its localization at the nuclear envelope (Fig. 2A-B″,C,D). We noticed, however, that Lamin C distribution was broader in myonuclei overexpressing BAF (Fig. 2C,D). Overexpression of BAF in control muscles did not affect BAF localization, nor did it affect Lamin C distribution (Fig. S2).

Taken together, these results show that BAF protein localization at the nuclear envelope depends on a functional LINC complex.

### The LINC complex is required for localization of the LEM-domain protein Otefin at the nuclear envelope

**Fig. 3.**
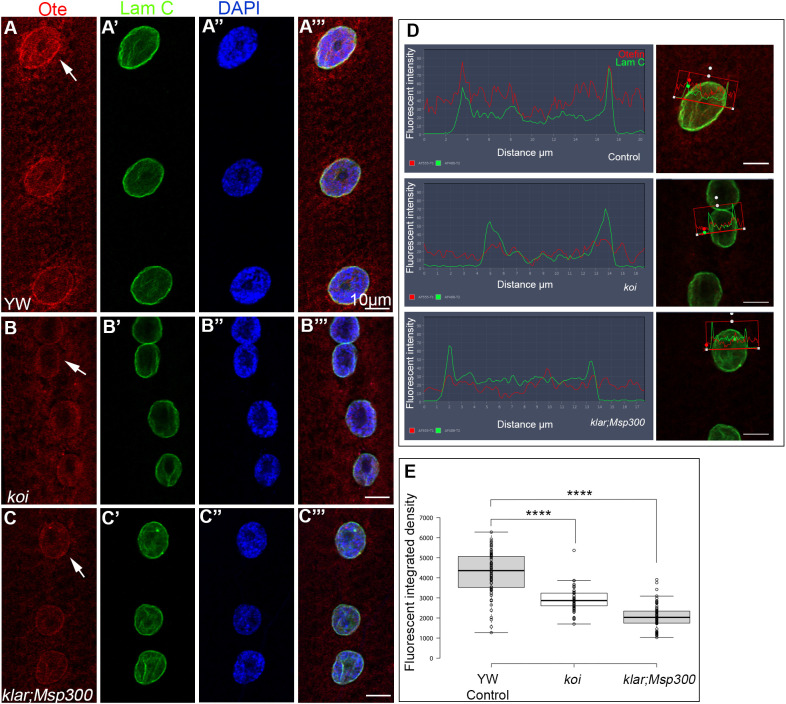
**Otefin levels at the nuclear envelope decrease in the LINC complex mutants.** (A-C‴) Representative larval muscle no. 7 of control (A-A‴), *koi* (B-B‴) and *klar;Msp300* mutants (C-C‴), labeled with anti-Otefin (red), anti-Lamin C (green) or DAPI (blue). Merged images are shown in A‴,B‴,C‴. (D) Line profiles of the nuclei indicated by arrows in A,B,C; the corresponding image of the nucleus analyzed is shown on the right of each profile. (E) Quantitative analysis of the fluorescence integrated density of Otefin for each group. The analysis was based on *n*=69 control nuclei, *n*=75 *koi* nuclei and *n*=72 *klar;Msp300* nuclei. A significant reduction in distribution of fluorescent Otefin at the nucleus (one-tailed *t-*test; ****P<0.0001) can be seen in the LINC mutants. Scale bars: 10 µm.

Previous studies indicated that BAF localization at the nuclear envelope is mediated by its binding to LEM-domain proteins (Cai et al., 2001; Mansharamani and Wilson, 2005). *Drosophila* has three proteins with a LEM domain, Otefin (Ote), Bocksbeutel (Bocks) and MAN1, which have been shown to function throughout *Drosophila* development, often redundantly (Barton et al., 2014). To address whether BAF exclusion from the nuclear envelope observed in the LINC complex mutants was downstream of a loss of LEM-domain proteins, we analyzed the localization of Otefin protein in the LINC mutant myonuclei. Labeling with anti-Otefin antibodies indicated its specific localization at the nuclear envelope of the myonuclei (Fig. 3A-A‴,D). Significantly, a substantial reduction in Otefin levels at the nuclear envelope was observed in myonuclei of both LINC mutants, *koi* and *klar;Msp300* (Fig. 3B-C‴,D,E). A significant difference between each mutant group and control was observed (*P*<0.0001). Assuming that BAF binds directly to Otefin (as described in other organisms), its decreased distribution at the nuclear envelope is downstream of both the LINC complex and Otefin.

### BAF localization at the nuclear envelope depends on association of nuclei with muscle sarcomeres

**Fig. 4.**
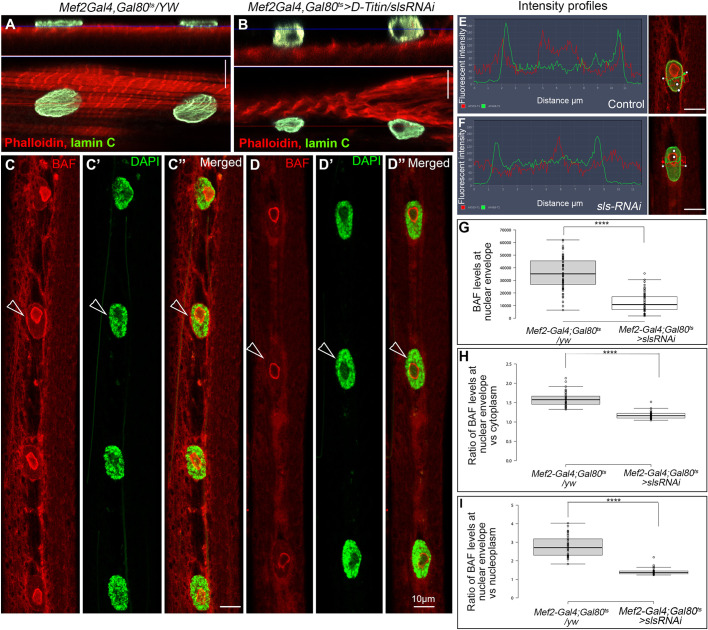
**Partial detachment of nucleus-sarcomere connections promotes the removal of BAF from the nuclear envelope.** (A-D″) Representative larval muscle no. 7 of control larvae (*Mef2Gal4, Gal80^ts^*; A,C-C″) or larvae in which *sls* was knocked down temporally at second instar stage (*Mef2Gal4, Gal80^ts^*>*sls*; B,D-D″). Labeling with phalloidin (red) and anti-Lamin C (green) in A,B indicates partial detachment of the nuclei from the sarcomeres and nuclear deformations (see orthogonal view in upper panels in A,B, in which the red line in each of the bottom panels indicates the position of the orthogonal optical section shown in the upper panels, and the blue line in the upper panels represents the position of the X-Y optical section shown in the bottom panels). (C,D) Labeling of BAF (red) and DAPI (green) in control larvae (C-C″) and larvae in which *sls* was temporally knocked down (D-D″)*.* Arrowheads indicate BAF at the nuclear envelope. Images A-D″ represent a single confocal Z stack. (E,F) Line profile of Lamin C and BAF fluorescence in myonuclei of control larvae (E) or larvae in which *sls* was temporally knocked down (F); corresponding nuclei are shown on the right. (G) Quantification of BAF fluorescence at the nuclear envelope of control larvae and larvae in which *sls* was temporally knocked down. Quantification was based on 72 control myonuclei and 66 myonuclei of larvae expressing *sls* RNAi. (H) Ratio between BAF fluorescence at the nuclear envelope and its levels in the cytoplasm in control larvae or in larvae in which *sls* was temporally knocked down. (I) Ratio between BAF fluorescence at the nuclear envelope and its levels in the nucleoplasm in control larvae or in larvae in which *sls* was temporally knocked down. Each of the experiments was repeated three times and indicated a similar trend. ****P<0.0001 (one-tailed *t-*test). Scale bars: 10 µm.

Our previous studies indicated that myonuclei associate with the muscle sarcomeres via a functional interaction between D-Titin (Sallimus or Sls) and Msp300 proteins (Elhanany-Tamir et al., 2012). In control larval preparations, myonuclear shape was ellipsoid at the X-Y axis, and extremely flat at the Z-axis (Fig. 4A). Temporal muscle-specific knockdown of *sls* (also known as *titin* or *D-titin*), induced in third instar larvae using RNA interference (*sls* RNAi) combined with *Gal4* and *Gal80^ts^* drivers, caused partial detachment of myonuclei from the sarcomeres. In these larvae, a significant deformation of nuclear morphology was observed, in which the nuclei become relatively more spheroid, indicative of changes in the mechanical environment of these nuclei (Fig. 4A,B) (Wang et al., 2015). Importantly, BAF dissociated specifically from the nuclear envelope in the *sls* knocked down muscles, whereas its localization around the nucleolus remained unchanged (Fig. 4C-D″,E,F). Measurement of BAF levels at the nuclear envelope (Fig. 4G), as well as quantification of the ratio between BAF levels at the nuclear envelope and those in the cytoplasm (Fig. 4H) or nucleoplasm (Fig. 4I), indicated a significant and specific decrease in BAF levels at the nuclear envelope. A statistically significant difference between the experimental group and control (*P*<0.0001) was observed. These results indicate that BAF localization at the nuclear envelope depends on proper connection between the myonuclei and sarcomeres.

### The serine/threonine kinase Ballchen regulates BAF localization at the nuclear envelope

**Fig. 5.**
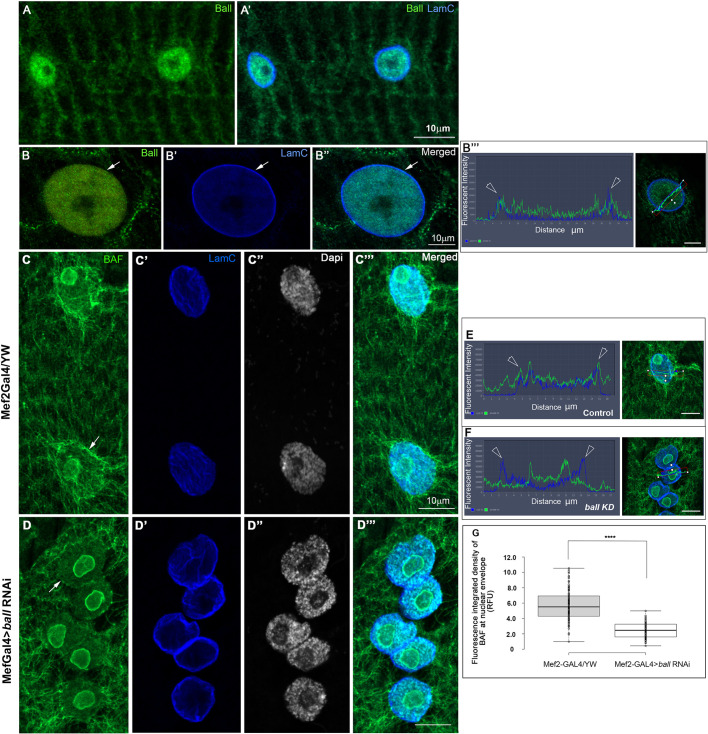
**BAF kinase Ball is required for BAF localization at the nuclear envelope.** (A-A′) Image of larval muscle no. 7 labeled with anti-Ball (green; A) and merged image of labeling with anti-Lamin C (blue) and anti-Ball (green) (A′). (B-B″) High resolution images of a control myonucleus labeled with anti-Ball antibody (green; B) or anti-Lamin C (blue; B′), and the merged image (B″), using expansion microscopy. Arrows indicate the nuclear envelope. (B‴) Line profile of the nucleus shown in B″ and in the right panel, with arrowheads indicating a peak for Ball labeling that overlaps with Lamin C. (C-D‴) Larval myonuclei from controls (*Mef2Gal4*/YW; C-C‴) and from muscles in which *ball* was knocked down by RNAi (*Mef2Gal4>ball RNAi*; D-D‴), labeled with anti-BAF (green), anti-Lamin C (blue) or DAPI (white). Merged images are shown in C‴,D‴. A decrease in the levels of BAF at the nuclear envelope following knockdown of Ball can be seen (arrows in C,D). (E,F) Line profiles of control (E) or *ball* knockdown (F) muscles with images of the corresponding nuclei on the right. Arrowheads show the Lamin C peaks (blue). (G) Quantification of BAF fluorescence integrated density at the outlines of the nuclear envelope indicates a significant decrease in BAF at the nuclear envelope (one-tailed *t-*test; ****P<0.0001). Quantification was based on *n*=118 control myonuclei and *n*=69 myonuclei from larvae expressing *ball* RNAi. Each of the experiments was repeated three times and indicated a similar trend. Images in all panels represent a single confocal Z stack. Scale bars: 10 µm.

**Fig. 6.**
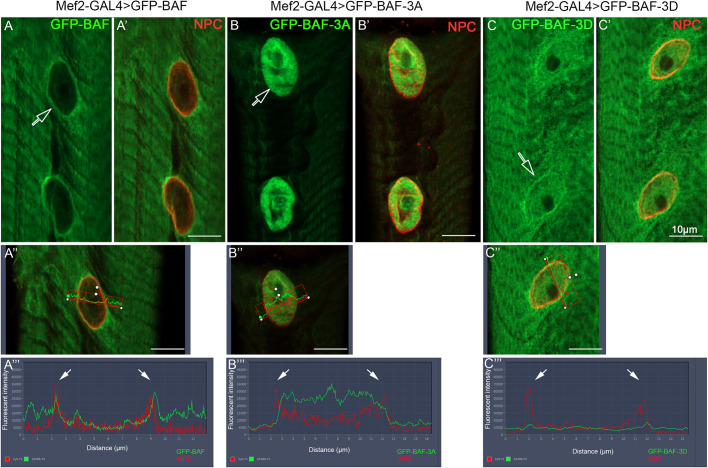
**GFP-BAF phospho-mutants localize differentially at the myonuclei.** Representative images of larval muscle no. 7 from larvae overexpressing GFP-BAF (A-A″), GFP-BAF-3A (non-phosphorylatable BAF; B-B″) or GFP-BAF-3D (phospho-mimicking BAF; C-C″), labeled with anti-GFP (green) and anti-NPC (FG repeats, red). (A‴,B‴,C‴) Line profiles of the images indicated by the corresponding empty arrows in A,B,C. Images of the corresponding nuclei are shown in A″,B″,C″. Solid arrows indicate the peaks for the nuclear membrane. Scale bars: 10 µm.

Previous reports indicate that BAF is phosphorylated by the nuclear serine/threonine kinase Ballchen (Ball), a homolog of vertebrate Vrk1 (Bengtsson and Wilson, 2006; Lancaster et al., 2007). We first analyzed the localization of Ball in *Drosophila* muscle fibers using specific antibodies (Herzig et al., 2014). Consistent with previous reports, Ball labeling was specifically observed within the myonuclei as well as along the sarcomeres (Fig. 5A,A′; Fig. S3). Fig. 5B-B″ shows co-labeling of Ball with Lamin C, using expansion microscopy (Chen et al., 2015; Jiang et al., 2018). This procedure allows a roughly fourfold increase in tissue size while preserving its 3D constituents, allowing high resolution imaging. A fine line of Ball labeling overlapped that of Lamin C (Fig. 5B-B″, arrows), indicating that in addition to its localization in the nucleoplasm, Ball is localized at the nuclear envelope. To address whether Ball controls the localization of BAF at the nuclear envelope, we knocked down *ball* (also known as *nhk-1*) in muscles using *ball* RNAi (Fig. S3 indicates the efficiency of the RNAi). Notably, reduction of Ball in the muscles led to a significant decrease in BAF localization at the nuclear envelope (Fig. 5C-D‴,E,F, quantified in Fig. 5G). The difference between the groups was statistically significant (*P*<0.0001). Interestingly, myonuclei of the *ball* knockdown muscles often exhibited an effect on nuclear position, partially phenocopying the nuclear phenotype of the LINC mutants. This implies that Ball is required for BAF localization at the nuclear envelope, presumably by promoting BAF phosphorylation. Furthermore, we performed muscle-specific expression of either GFP-BAF, phospho-mutant BAF, in which the phosphorylated serine-threonine residues in its N-terminal end are mutated to alanine (GFP-BAF-3A), or phospho-mimic GFP-BAF (GFP-BAF-3D) (Hou et al., 2016). The GFP-BAF was localized at the nuclear membrane and also in the cytoplasm, similarly to endogenous BAF, (Fig. 6A-A‴). However, the non-phosphorylatable version of BAF (GFP-BAF-3A) was eliminated from the nuclear envelope and accumulated in the nucleoplasm (Fig. 6B-B‴). Importantly, 100% of the larvae expressing the non-phosphorylatable GFP-BAF-3A form were extremely sick and did not develop further into the pupal stage. The phospho-mimicking BAF (GFP-BAF-3D) did show a specific localization of the GFP at the nuclear envelope and increased levels in the cytoplasm and the nucleoplasm (Fig. 6C-C‴). The requirement of Ball kinase for BAF localization at the nuclear envelope, and elimination of the phospho-mutant BAF from the nuclear envelope, indicate that BAF phosphorylation is crucial for its localization at the nuclear envelope.

### Detachment of BAF from the nuclear envelope correlates with increased DNA levels in the myonuclei

**Fig. 7.**
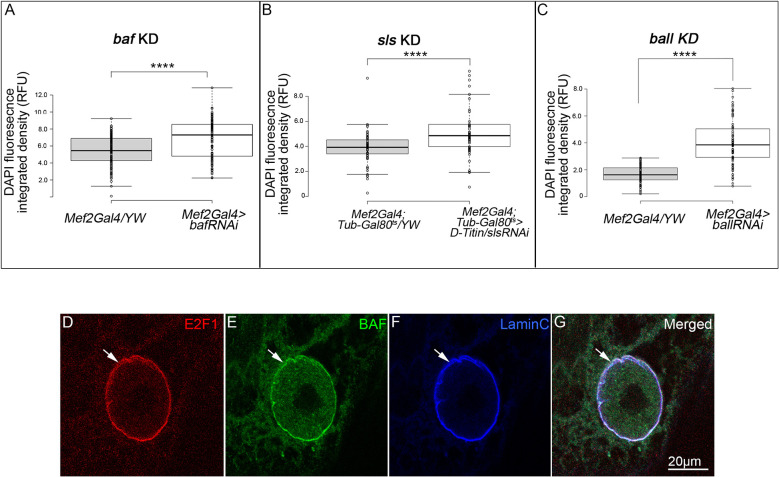
**Removal of BAF from the nuclear envelope correlates with increased DNA levels in the myonuclei.** (A-C) Quantification of the DNA content per myonucleus performed by measuring DAPI integrated density in myonuclei following muscle-specific knockdown of *baf* (A) (*n*=150 control myonuclei and *n*=106 myonuclei from *baf* RNAi), *sls* (B) (*n*=72 control myonuclei and *n*=66 myonuclei from *sls* RNAi) and *ball* (C) (*n*=118 control myonuclei and *n*=69 nuclei from *ball* RNAi). The difference between the experimental groups and controls is statistically significant; ****P<0.0001 (one-tailed *t*-test). Each of the experiments was repeated three times and indicated a similar trend. (D-G) High resolution images of the nuclear localization of E2F1 (D), BAF (E) and Lamin C (F) in myonuclei using expansion microscopy. Merged images are shown in G. Arrows indicate colocalization of E2F1, BAF and Lamin C at the nuclear envelope. Scale bar: 20 µm.

Previously we demonstrated that LINC mutant muscles exhibit an increase in DNA endoreplication, leading to elevated DNA content in the myonuclei (Wang et al., 2018). We wished to address whether the localization of BAF at the nuclear envelope correlates with increased endoreplication and elevated DNA content in the myonuclei. To this end, we quantified the DNA content in myonuclei of *baf*,* sls* and *ball* knocked down muscles. The larvae in each of these experiments were staged, fixed and labeled with Lamin C and DAPI, in parallel to control larvae. Quantification of the DNA content indicated that the DNA content increased in all three experimental conditions, relative to controls (Fig. 7A-C; *P*<0.0001 for each experiment). Of note, the *baf* RNAi line that we used led to reduction in levels of BAF protein and *baf* mRNA (Fig. S1E-I). Furthermore, the reduction in BAF was observed in all subcellular localizations, including the cytoplasm, nuclear envelope and nucleolus (Fig. S1E-H). We followed the viability and developmental timing of 50 staged embryos expressing *baf* RNAi in the muscles and compared them with controls. No detectable defects in larval developmental timing nor in the percentage of flies that eclosed relative to control were observed, suggesting that the elevation in DNA content in the larval muscles does not abrogate muscle function.

For the *sls* RNAi larvae (Fig. 7B), driven by *Mef2Gal4* combined with *Gal80^ts^*, larvae were raised at permissive temperature up to second instar stage and then transferred to a restrictive temperature to allow temporal expression of the RNAi at third instar larval stage. Taken together, these results suggest a functional correlation between dissociation of BAF from the nuclear envelope and an increase in the DNA content of the myonuclei.

To reveal a possible molecular explanation for the correlation between BAF localization at the nuclear membrane and DNA endoreplication, we focused on E2F1, a key transcription factor that regulates DNA endoreplication in flies (Zielke et al., 2011). Expansion microscopy was used to analyze the fine subcellular localization of E2F1 in the myonuclei at high resolution. Notably, we found that E2F1 accumulated at the nuclear envelope, overlapping BAF and Lamin C (Fig. 7D-G). BAF labeling at the nucleolus borders was lost in this method, possibly due to the procedure used for expansion microscopy. The specific localization of E2F1 at the nuclear envelope, and its overlapping distribution with BAF, suggests a functional coupling between both proteins.

### Increased levels of E2F1 in the nucleoplasm correlate with decreased BAF at the nuclear envelope

**Fig. 8.**
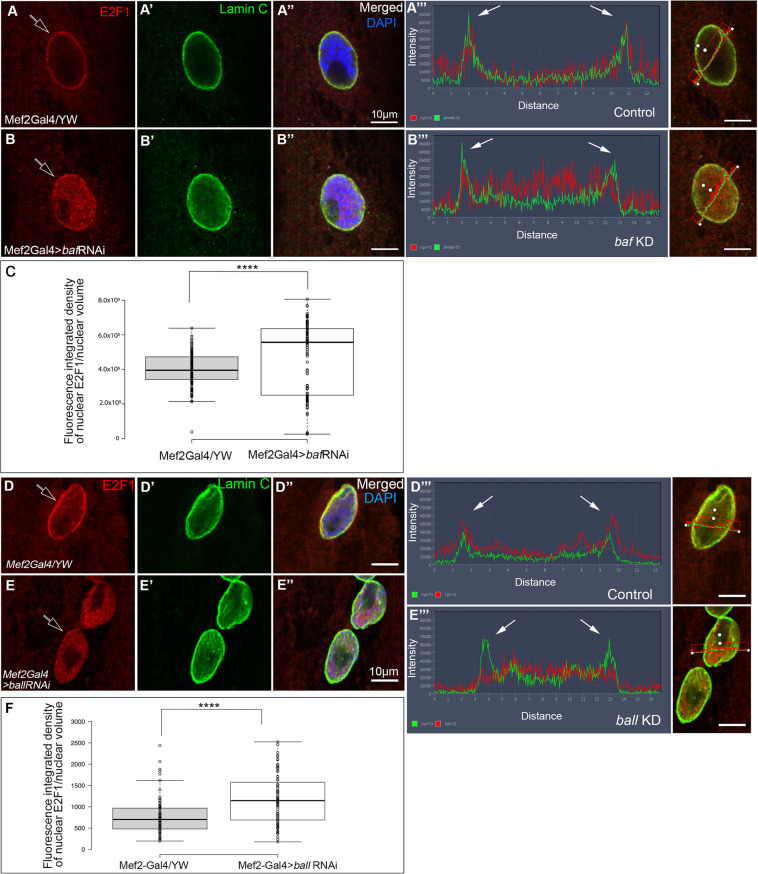
**Levels of E2F1 in the nucleoplasm increase following knockdown of *baf* or *ball*.** (A-A″,B-B″) Myonuclei from control (A-A″) and *baf* knockdown (KD) (B-B″) muscles labeled with anti-E2F1 (red) or anti-Lamin C (green). Merged images are also labeled with DAPI (blue). Empty arrows indicate the nuclear envelope. (A‴,B‴) Line profiles of E2F1 (A‴) and Lamin C (B‴); corresponding nuclei are shown on the right. Note the specific localization of E2F1 at the nuclear envelope in controls and its enrichment in the nucleoplasm in *baf* KD muscles. (C) Quantification of the integrated density of E2F1 fluorescence in the entire nuclear volume indicates a significant increase in E2F1 levels in the nucleoplasm of *baf* KD myonuclei. Quantification was based on *n*=150 control myonuclei and *n*=106 myonuclei from *baf* KD larvae. (D-D″,E-E″) Myonuclei from control (D-D″) and *ball* KD muscles (E-E″) labeled with anti-E2F1 (red) or Lamin C (green). Merged images are also labeled with DAPI (blue). (D‴,E‴) Line profiles of E2F1 and Lamin C indicate the specific localization of E2F1 at the nuclear envelope in controls and its accumulation in the nucleoplasm of *ball* KD muscles; corresponding nuclei are shown on the right. (F) Quantification of the fluorescence integrated density of E2F1 levels in the entire nuclear volume of *ball* KD muscles indicates a significant increase in E2F1 levels in *ball* KD myonuclei. Quantification was based on *n*=100 control myonuclei and *n*=126 myonuclei from larvae expressing *ball* RNAi. In C and F, ****P<0.0001 (one-tailed *t*-test). Empty arrows indicate E2F1 levels in the nucleoplasm. Filled arrows indicate the nuclear envelope borders. Scale bars: 10 µm.

Next, we analyzed the levels of E2F1 in myonuclei in which BAF levels were knocked down by RNAi. Notably, E2F1 levels were significantly higher in the nucleoplasm of myonuclei in larvae expressing *baf* RNAi (Fig. 8, compare B-B‴ with control A-A‴, quantified in C). A statistically significant increase in E2F1 nuclear levels was observed in the *baf* RNAi larvae relative to controls (*P*<0.0001). These results suggest an inhibitory function for BAF on the nuclear accumulation of E2F1, which is consistent with the increase in DNA content in *baf* knockdown myonuclei.

Next, we analyzed the levels of E2F1 in the nucleoplasm in *ball* knocked down muscles. Significantly, E2F1 levels were specifically elevated in the myonuclei, concomitant with a decrease in the levels of E2F1 at the nuclear envelope (Fig. 8, compare E-E‴ with control in D-D‴, quantified in F). The difference between the groups was statistically significant (*P*<0.0001). These results are consistent with an inhibitory function for Ball on E2F1 nuclear levels.

**Fig. 9.**
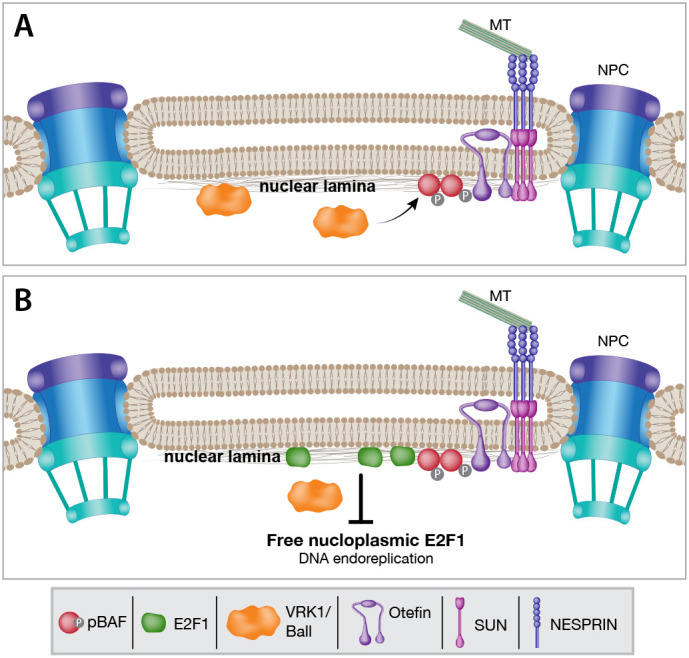
**Proposed model for the coupling between LINC-dependent localization of BAF at the nuclear envelope and endoreplication.** (A) The LINC complex protein Nesprin associates with the microtubule cytoskeleton at its N-terminal end and binds to SUN domain proteins at the perinuclear space via its KASH domain at the C-terminal end. The LINC complex maintains the localization of the LEM-domain protein Otefin at the nuclear envelope. Otefin recruits and binds to phosphorylated BAF, thus promoting BAF localization at the nuclear envelope. BAF phosphorylation is induced by the kinase Ball, which is found at both the nucleoplasm and nuclear envelope. (B) E2F1 accumulates at the nuclear envelope through BAF at the nuclear envelope. E2F1 accumulation at the nuclear envelope lowers its free increase in the nucleoplasm. Decreased E2F1 levels in the nucleoplasm attenuate cell cycle progression and DNA endoreplication. MT, microtubule; NPC, nuclear pore complex.

In summary, our results suggest a model in which, in mature non-dividing muscle fibers, the LINC complex promotes proper localization of the LEM protein Otefin at the nuclear envelope. Ball kinase phosphorylates BAF, promoting its binding to Otefin (Fig. 9A). BAF at the nuclear envelope is essential for accumulation of E2F1 at the nuclear envelope and for reducing its levels in the nucleoplasm (Fig. 9B). Assuming that E2F1 is a limiting factor in promoting endoreplication in muscles, its increase in the nucleoplasm would promote DNA synthesis (endoreplication) in the myonuclei.

## DISCUSSION

Endoreplication is used by differentiated cells as a strategy for growing in size, and to accommodate tissue injury. Coupling between the endoreplication process and the mechanical environment of the cell allows the nucleus to respond to mechanical changes following tissue injury without a need for cell division. This is especially true in myofibers, where reiterated mechanical inputs are applied on the nuclear envelope during muscle contractile waves. Here, we demonstrate the contribution of a novel mechanosensitive component, BAF, in controlling the nuclear accumulation of E2F1, a crucial transcription factor required for the regulation of endoreplication (Huang et al., 2005; van den Heuvel and Dyson, 2008; Zielke et al., 2011). Whereas previous reports implicated BAF in promoting the condensation and assembly of post-mitotic dsDNA into single nuclei (Samwer et al., 2017), here we demonstrate that BAF is also essential for the arrest of DNA endoreplication in fully differentiated muscle fibers. Importantly, only BAF that localizes to the nuclear envelope appears to be relevant for this function in post-mitotic differentiated cells. The contribution of BAF to larval muscle functionality is unclear, as *baf* mutants did not survive up to third instar stage and BAF knockdown in muscles by using RNAi did not eliminate BAF very efficiently.

In *Drosophila* muscle fibers, we found that BAF was detected in various subcellular sites, including the cytoplasm, nuclear envelope, nucleoplasm and at the nucleolus borders. Yet, only the portion of BAF localized at the nuclear envelope was found to change following elimination of a functional LINC complex. It is well accepted that the LINC complex transmits cytoplasmic mechanical inputs from the cytoskeleton to the nucleoskeleton in various cell types (Chambliss et al., 2013; Guilluy et al., 2014). Moreover, nuclear deformations (from oval into spheroid shape) observed both in larval muscles of LINC complex mutants (Elhanany-Tamir et al., 2012; Wang et al., 2015) and in conditions where nuclei detach from the sarcomeres [e.g. Wang et al. (2015) or following Sls knockdown] are indicative of changes in the mechanical inputs applied on the nuclear envelope. Because BAF localization at the nuclear envelope was specifically impaired in both conditions, we propose that maintenance of BAF at the nuclear envelope is mechanically sensitive.

In control myofibers, BAF exhibited a relatively broad distribution along the outlines of the nuclear envelope, often extending beyond the Lamin C expression domain towards the cytoplasm, overlapping with the nucleus-associated microtubules (Fig. S1C,D). This suggested that, in addition to its association with the inner aspects of the nuclear membrane through binding to LEM-domain proteins and Lamin A/C (Cai et al., 2001; Jamin and Wiebe, 2015; Shimi et al., 2004; Wilson et al., 2005), BAF associates with the outer aspects of the nuclear membrane. Previous experiments indicate that despite its small size BAF does not diffuse passively from the cytoplasm to the nucleus (Shimi et al., 2004). Furthermore, photobleaching experiments with GFP-BAF indicate that BAF-dependent repair of nuclear ruptures occurs when cytoplasmic BAF, but not nuclear BAF, rapidly associates with the ruptured sites and further recruits LEM-domain proteins to establish membrane sealing (Halfmann et al., 2019). The authors suggest that their findings are consistent with a dynamic exchange of BAF between cytoplasmic and nuclear pools, where BAF in the cytoplasm primarily responds to mechanical signals. Because our experiments indicate that BAF phosphorylation is crucial for its maintenance at the nuclear membrane, it is possible that the exchange of BAF localization between the cytoplasm and the nucleus is stabilized by its phosphorylation. The contribution of the LINC complex to BAF association with the nuclear envelope could be either direct (e.g. by binding to components of the LINC complex) or indirect (e.g. through an effect of the LINC complex on the distribution of LEM proteins at the nuclear envelope). Our results support the latter model, in which the LINC complex maintains the localization of the LEM protein Otefin at the nuclear envelope to mediate BAF association with the nuclear envelope. Hence, we suggest a model in which the contribution of the LINC complex to BAF localization at the nuclear envelope is through an effect on Otefin localization at the nuclear envelope (see Fig. 9).

Endoreplication has been implicated in a wide variety of differentiated cells in a broad range of species, including human tissues (Edgar and Orr-Weaver, 2001; Fox and Duronio, 2013; Gan et al., 2019; Lee et al., 2009; Losick et al., 2013; Zielke et al., 2013). A link between mechanical tension and endoreplication has been recently suggested (Cao et al., 2017). However, the molecular mechanism coupling mechanical tension with the endoreplication process is still elusive. Here, we found that a key regulator of endoreplication, E2F1, exhibits a specific distribution at the nuclear envelope in fully differentiated myofibers, where it probably resides non-actively. Changes in the mechanical environment of the nuclear envelope correlate with the localization of E2F1 and promote its accumulation within the nucleoplasm, where it is expected to promote DNA synthesis. It will be of interest to find which proteins associate directly with E2F1 at the nuclear envelope. Our attempts to co-immunoprecipitate BAF with Msp300 or E2F1 failed to show a specific protein interaction between these proteins. From a physiological point of view, no detectable changes in muscle size or movement were observed in the *baf* knockdown muscles, and the larvae developed up to adult stage. The *baf* homozygous mutant did not develop up to the third instar larval stage, so the full physiological contribution of BAF to muscle growth awaits experiments in which a more efficient reduction in BAF levels is induced in muscle tissue.

In summary, our results reveal a novel insight into the role of the LINC complex in coupling endoreplication with changes in the nuclear envelope composition in mature muscle fibers. In particular, the mechanosensitive component, BAF, whose localization at the nuclear envelope is tightly regulated by the LINC complex, is shown to negatively control the nuclear accumulation of the cell cycle regulator E2F1 at the level of the nuclear envelope. The localization of Otefin in the nuclear envelope and BAF phosphorylation by Ball kinase are both crucial in this context. This process might be part of a mechanosensitive pathway that regulates polyploidy in a wide variety of differentiated cells.

## MATERIALS AND METHODS

### Fly stocks and husbandry

All crosses were performed at 25°C and flies were raised on cornmeal yeast agar. The following stocks were distributed by the Bloomington Drosophila Stock Center: tubP-*Gal80^ts^*/TM2 (FBst0007017), *Gal4-*Mef2.R (FBst0027390), *baf* RNAi (FBst0036108), *sls* RNAi (FBgn0086906), *ball* RNAi (FBti0130755). Ball-GFP was obtained from the Vienna BioCenter (VDRC): FlyFos016090 (pRedFlp-Hgr)(ball[41529]::2XTY1-SGFP-V5-preTEV-BLRP-3XFLAG)dFRT. UAS-GFP-BAF, UAS-GFP-BAF-3A (obtained from Lei Liu, Beijing Institute for Brain Disorders, Beijing, China). *klar^ΔKASH(mCD4)^* and *Msp300^ΔKASH^* (J.A. Fischer, University of Texas, Austin, TX) were combined on Cyo-YFP and TM6Tb balancers, *koi^84^*(FBst0025105)/Cyo-dfd-eYfp (Kracklauer et al., 2007).

Staging of the larvae was performed by 6 h embryo collection and further growth on yeast paste in vials up to early third instar stage. Temporal expression of *sls* RNAi or GFP-BAF, as well as GFP-BAF-3A, was performed using a combination of *Mef2Gal4* and tubGal80^ts^ drivers as follows: embryo collection was performed at 25°C for 6 h, followed by transfer to a permissive temperature of 18°C up to first instar larval stage. Then, larvae were transferred to the restrictive temperature of 29°C up to early third instar stage.

### Immunofluorescence

Immunofluorescence staining was performed as previously described (Wang et al., 2015). For fixation, paraformaldehyde (4% from 16% stock of EM grade; #15710; Electron Microscopy Sciences) was used without methanol to avoid damage to native F-actin or chromosomal morphology. Specimens were fixed for approximately 30 min and subsequently washed several times in PBS containing 0.1% Triton X-100 (PBST) on a horizontal shaker with gentle agitation. Image analyses were consistently performed on muscle 7. All specimens were mounted in Shandon ImmuMount for microscopy (ThermoFisher Scientific). Control and experiment larvae were staged and grown in parallel time intervals. Fixation and antibody staining of control and experiment larvae were done in the same tube, marking one group by head excision.

### Antibodies and synthetic dyes

Mouse anti-Lamin C (DSHB, no. LC28.26-c) was obtained from the Developmental Studies Hybridoma Bank, created by the NICHD of the NIH and maintained at The University of Iowa. Rat anti-α-Tubulin (1:200 dilution; Bio-Rad MCA78G), chicken anti-GFP (1:200 dilution; Abcam #13970), rabbit anti-BAF [1:300 dilution; provided by Paul Fisher, Stony Brook, NY, and Ryszard Rzepecki, University of Wroclaw, Poland (Furukawa et al., 2003)] and rat anti-E2F1 [1:200 dilution; provided by Stefan Thor, University of Queensland, Australia (Baumgardt et al., 2014) and Jonathan Benito-Sipos, University of Madrid] were used for immunofluorescence. Rabbit anti-Otefin (1:300 dilution) was provided by Y. Gruenbaum (Hebrew University, Jerusalem, Israel). Secondary antibodies used were Alexa Fluor 488, 555 and 647 conjugated secondary antibodies against rat, chick, rabbit and mouse (1:300 dilution), purchased from The Jackson Laboratory and ThermoFisher Scientific.

For labeling of chromatin, we used DAPI (1 µg/ml; Sigma-Aldrich). For F-actin labeling, we used TRITC-phalloidin (Sigma-Aldrich P1951). Labeling was performed by exposing the larvae from experimental and control groups to a similar antibody mix in the same tube.

### Expansion microscopy

The procedure was essentially as described (Jiang et al., 2018). Briefly, larvae were fixed with 4% PFA in PBS, washed with PBST, blocked with 10% BSA in PBST, incubated with first antibody overnight, and with secondary antibodies (conjugated with Atto 647, Alexa Fluor 555 or Alexa Fluor 488) overnight. After washing with PBST and PBS, the larvae were incubated with 1 mM MA-NHS in PBS for 1 h, washed with PBS, incubated with monomer solution (2 M NaCl, 8.625% sodium acrylate, 2.5% acrylamide, 0.15% bisacrylamide, 10× PBS) for 45 min at 4°C, and then transferred to gelation solution (0.2% TEMED, 0.01% TEMPO, 95% monomer solution and 0.2% APS) for 30 min at 37°C. The gel with the larvae was incubated with chitinase (1 Unit/ml) in PBS pH 6.0 for 4 days at 37°C. Three washes with PBS were followed by addition of collagenase (1 mg/ml) in 1× HBSS (with 0.01 M CaCl_2_ and 0.01 M MgCl_2_) and incubation at 37°C overnight. Washes with PBS were followed by addition of Proteinase K (8 Units/ml) in digestion buffer, incubation for 1 h at 37°C and additional washes with PBS. Hoechst dye was added for 10 min and the samples washed. Expansion was performed by the addition of ultrapure water for 30 min. The sample was then imaged under the confocal microscope.

### Microscopy and image analysis

Microscopy images were acquired at 23°C using confocal microscope Zeiss LSM 800 with the following lenses: Zeiss C-Apochromat 40×/1.20 W Korr M27 and 20× PlanApochromat 20/0.8. The microscopy samples were embedded with Coverslip High Precision 1.5 H±5 μm (Marienfeld-Superior, Lauda-Königshofen, Germany). Immersion medium Immersol W 2010 (ne=1.3339) and immersion oil Immersol 518 F (ne=1.518) were used. Images were analyzed with Fiji, including plug-ins and adapted scripts. Figure panels were finally assembled using Photoshop CC 2019. Acquisition software was Zen 2.3 (blue edition).

### Data collection

Quantification of fluorescence integrated density (amount of total fluorescence per cell) of proteins in myonuclei was performed using a custom-built FIJI macro, which included rolling ball background subtraction. The macro has been deposited in GitHub (doi:10.5281/zenodo.3372266). In brief, Lamin C was used to define the entire nuclear volume as the region of interest (ROI). For each myofibril, fluorescence integrated density in different channels was measured in all the slices along the Z-direction of the ROI. This allowed us to measure the levels of specific proteins inside the myonuclei in an unbiased manner. To measure BAF levels along the nuclear envelope, we used the manual selection tool for the Lamin C channel to define the nuclear membrane as the ROI. The fluorescence integrated density of BAF along the Z-direction of the ROI was then analyzed. To assess BAF localization at the nuclear envelope in each myofibril, the nuclear envelope of myonuclei was marked using the ImageJ composite selection tool for the Lamin C channel to define the nuclear envelope as the ROI; fluorescence integrated density was measured in all the slices along the Z-direction of the ROI. The ratio of BAF along the cytoplasm and nucleoplasm with respect to the nuclear envelope was measured using the line profile tool (Zeiss, Zen). BAF fluorescence at the nuclear envelope, cytoplasm and nucleoplasm was measured along a line profile in a single, middle Z section.

### Statistical analysis

Statistical analysis was performed using GraphPad Prism and Microsoft Excel 2016. Measurements were analyzed using a two-independent samples *t*-test. *P*-values <0.05 were considered statistically significant. BoxPlotR was used to generate box plots (Spitzer et al., 2014), in which the center lines represent the medians, box limits indicate 25th and 75th percentiles, crosses represent sample means and dots represent outliers (data points beyond the 95% confidence intervals). Furthermore, whiskers, determined using the Tukey method, extended to data points less than 1.5 interquartile ranges from the first and third quartiles as determined by the BoxPlotR software. All experiments were repeated three times independently and in at least four or five randomly selected larvae from which muscle no. 7 was monitored in at least four abdominal segments per experimental group.

### RT-qPCR

Gene expression quantification was carried out by reverse transcription quantitative real time PCR (RT-qPCR). Briefly, total muscle RNA was isolated from larval body walls of 30 dissected *Drosophila* individuals using RNeasy Protect Mini Kit (Qiagen). A sample of total RNA (1 μg) was used for first strand cDNA synthesis by reverse transcription using SuperScript Reverse Transcriptase IV (Invitrogen) with oligonucleotide dT primers. qPCR was performed using gene-specific primers on the ABI 7500 Real-Time PCR System (Applied Biosystems) with Fast SYBR Green Master Mix (Applied Biosystems) for detection. Each sample was run in triplicate. The primer sequences are listed in Table S1. The difference in gene expression was calculated using the fold-change (ΔΔCt method) (Schmittgen and Livak, 2008). ΔCt is the Ct value for the gene of interest normalized to the Ct value of the respective gene in both control larvae (armadillo-*Gal4*/YW) and BAF knockdown larvae (armadillo-Gal4>*baf* RNAi). ΔΔCt values were calculated as a relative change in ΔCt of the target gene in BAF knockdown with respect to control (house-keeping gene *succinate dehydrogenase*). Fold changes were expressed as 2−ΔΔCt for upregulated genes and the negative reciprocal of the fold change for downregulated genes (where 2−ΔΔCt<1).

## Supplementary Material

Supplementary information

Reviewer comments
