# Supplementary material for: Recruitment of BAF to the nuclear envelope couples the LINC complex to endoreplication
Source: Development. 2020 Dec 13;147(23):dev191304. doi: 10.1242/dev.191304 (PMC7758627; doi:10.1242/dev.191304)
Supplement: Supplementary information [file develop-147-191304-s1.pdf]

Supplement Figures

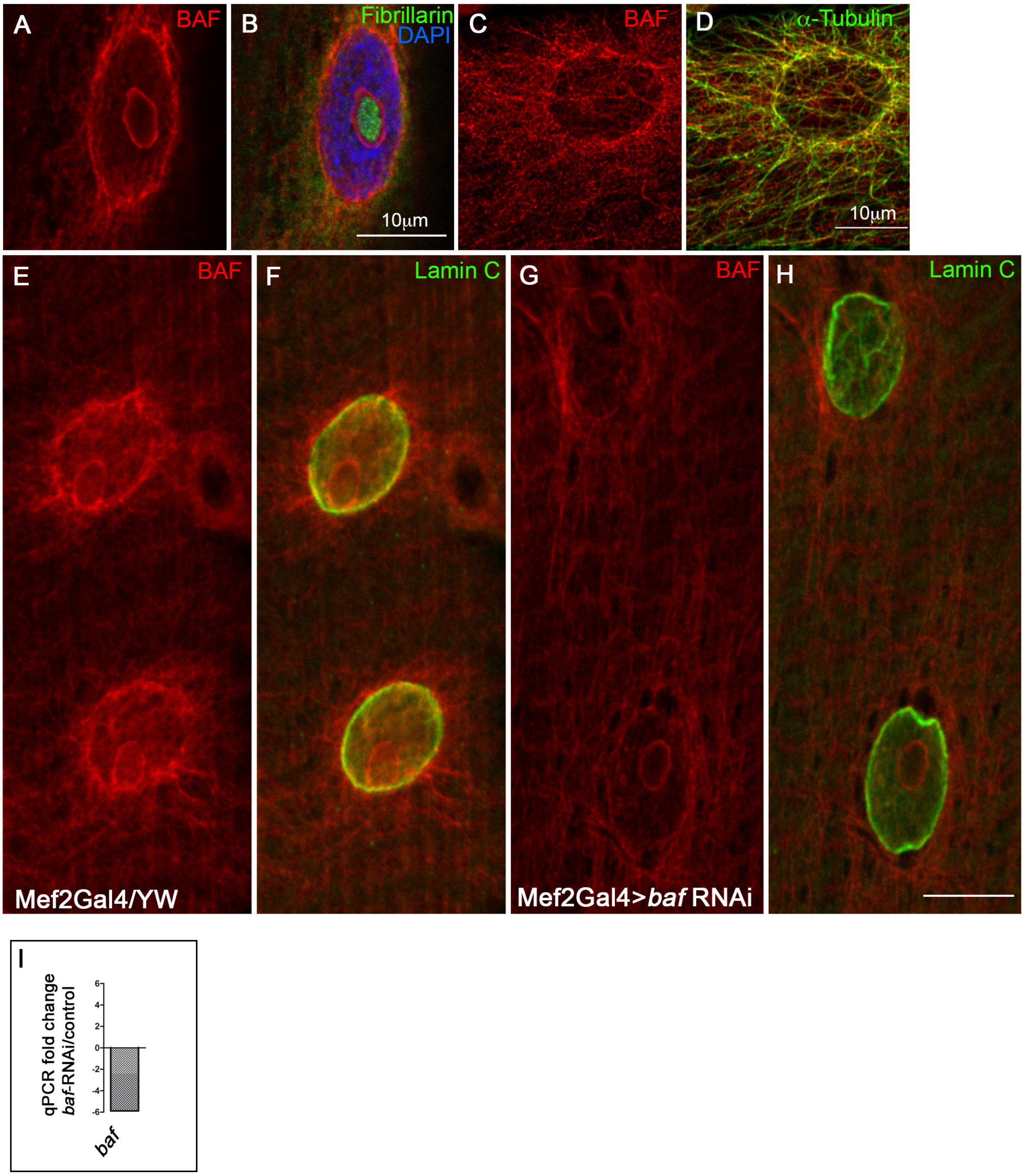

Figure S1: Evaluation of BAF antibody specificity

A single myonucleus labeled with anti BAF (red, A), and (B) a merged image of this nucleus labeled with BAF (red) Fibrilarin (green), and DAPI (blue). A single myonucleus labeled with BAF (red, C) and (D) a merged image of this nucleus labeled with BAF (red), α-Tubulin (green) and DAPI (blue).

Two nuclei of larval muscle (no. 7) labeled with anti BAF (red,E-H), or their merged images together with lamin C (green F, H), of control (Mef2Gal4/YW), or *baf*-RNAi (Mef2Gal4>*baf*-RNAi) larvae. Note a specific reduction in BAF labeling. I) qPCR analysis using *baf* primers, as well as with primers for house-keeping gene succinate dehydrogenase (SDH) (for normalization), of control larvae (armGal4/YW), or *baf* RNAi larvae (armGal4>*baf*-RNAi). This analysis indicated a 5 fold reduction in *baf* mRNA levels.

Development • Supplementary information

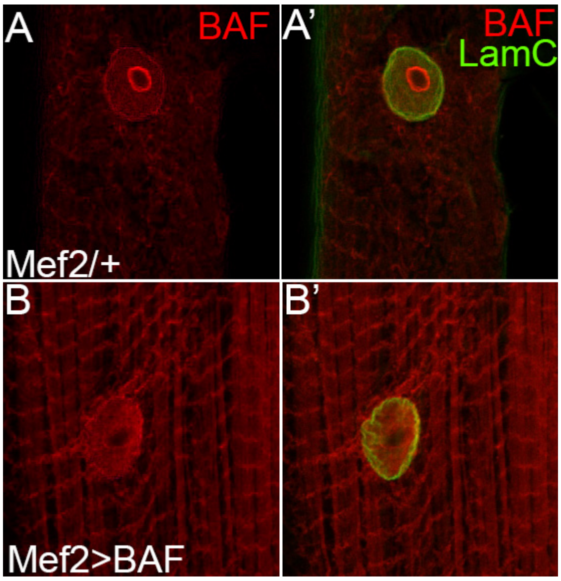

Figure S2: Overexpression of BAF in muscles does not alter its localization at the nuclear envelope

Muscle nuclei from control larvae (MefGal4/YW, A, A') or larvae overexpressing BAF (B, B'), labeled with anti BAF (red, A, B), or their merged images with lamin C (A', B' green). BAF subcellular localization does not change, following its overexpression in muscles.

Development • Supplementary information

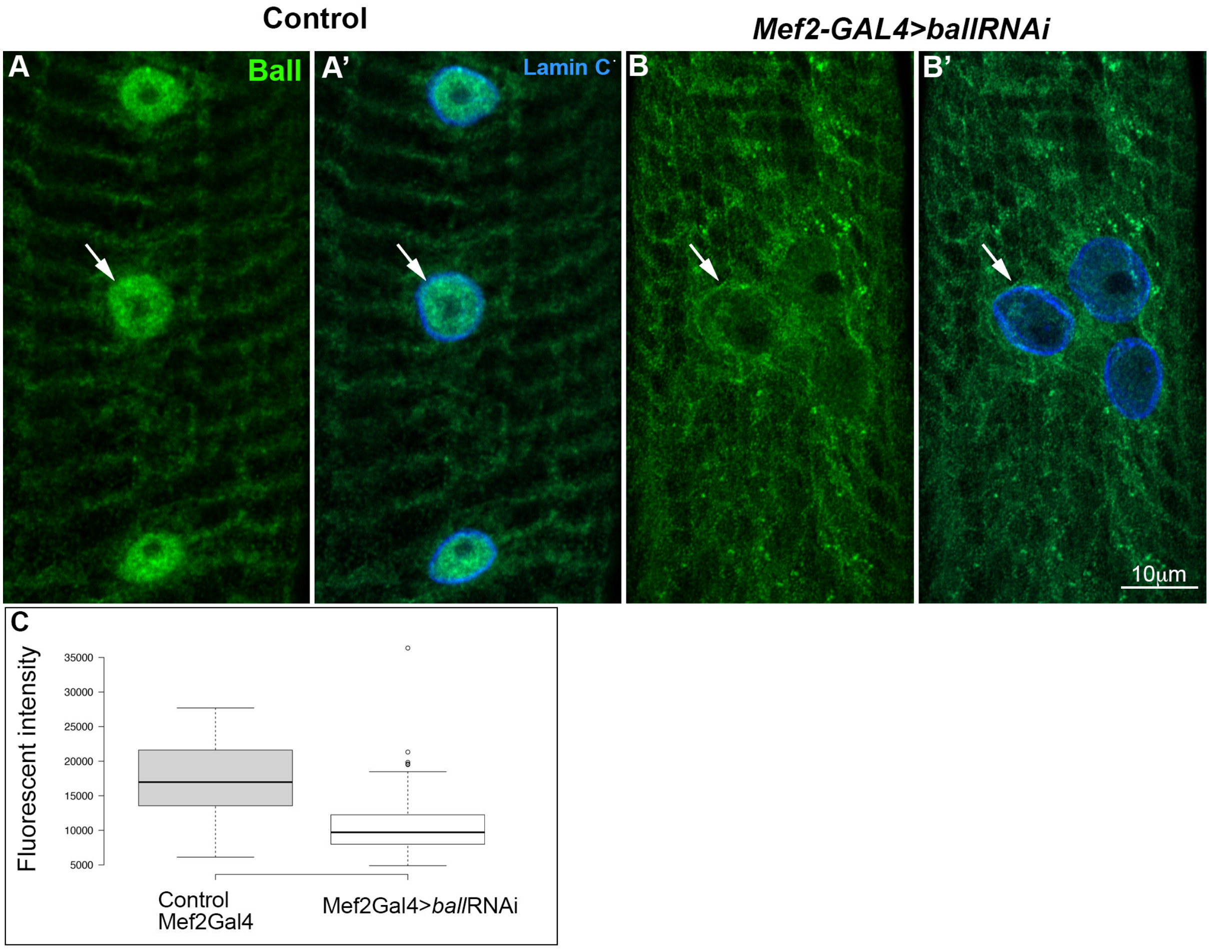

Figure S3: Specificity of Ball antibody labeling

Myonuclei form control larvae labeled with anti Ball antibody (green, A) and Lamin C (blue, A') indicating a specific localization of Ball in the myonuclei. Larvae expressing *ball* RNAi in muscles and labeled with anti Ball (green, B), and with Lamin C (blue, B'). A significant reduction of Ball labeling in the myonuclei is demonstrated. C – Quantification of the fluorescence of Ball relative to control.

Table S1: List of primers used for RT-qPCR

Gene	RT-qPCR primers
baf	F: 5'- AACACAGGAACTTCGTTGCG - 3'<br>R: 5'- AGAACTCCTCGCACCAATCG - 3'
SdhA	F: 5'- ACCAACACCAAACACCCCAT - 3'<br>R: 5'- CACGTTGCATGGAGGCGA - 3'

Development • Supplementary information
