# Supplementary material for: Recruitment of BAF to the nuclear envelope couples the LINC complex to endoreplication
Source: Development. 2020 Dec 13;147(23):dev191304. doi: 10.1242/dev.191304 (PMC7758627; doi:10.1242/dev.191304)
Supplement: Reviewer comments [file develop-147-191304_review_history.pdf]

Recruitment of BAF to the nuclear envelope couples the LINC complex to endoreplication

C.P. Unnikannan, Adriana Reuveny, Dvorah Grunberg and Talila Volk
DOI: 10.1242/dev.191304

Editor: Thomas Lecuit

Review timeline

Original submission:	6 April 2020
Editorial decision:	8 June 2020
First revision received:	20 July 2020
Editorial decision:	11 September 2020
Second revision received:	13 September 2020
Editorial decision:	27 October 2020
Third revision received:	27 October 2020
Accepted:	29 October 2020

Original submission

First decision letter

MS ID#: DEVELOP/2020/191304

MS TITLE: Recruitment of BAF to the nuclear envelope couples nuclear mechanics with endoreplication

AUTHORS: C.P. Unnikannan, Adriana Reuveny, Dvorah Grunberg, and Talila Volk

I apologize for the delay before coming back to you. I have now received all the referees' reports on the above manuscript, and have reached a decision. The referees' comments are appended below, or you can access them online: please go to BenchPress and click on the 'Manuscripts with Decisions' queue in the Author Area.

As you will see, the referees express strong interest in your work, but two of them in particular raises a number of points and recommend a substantial revision of your manuscript before we can consider publication. While not necessarily agreeing with all the comments, I feel the referees make a number of very constructive comments that should strengthen the conclusions of your manuscript. If you are able to revise the manuscript along the lines suggested, which may involve further experiments, I will be happy receive a revised version of the manuscript. Your revised paper will be re-reviewed by one or more of the original referees, and acceptance of your manuscript will depend on your addressing satisfactorily the reviewers' major concerns. Please also note that Development will normally permit only one round of major revision.

We are aware that you may currently be unable to access the lab to undertake experimental revisions. If it would be helpful, we encourage you to contact us to discuss your revision in greater detail. Please send us a point-by-point response indicating where you are able to address concerns raised (either experimentally or by changes to the text) and where you will not be able to do so within the normal timeframe of a revision. We will then provide further guidance. Please also note that we are happy to extend revision timeframes as necessary.

© 2020. Published by The Company of Biologists under the terms of the Creative Commons Attribution License (https://creativecommons.org/licenses/by/4.0/).

Please attend to all of the reviewers' comments and ensure that you clearly highlight all changes made in the revised manuscript. Please avoid using 'Tracked changes' in Word files as these are lost in PDF conversion. I should be grateful if you would also provide a point-by-point response detailing how you have dealt with the points raised by the reviewers in the 'Response to Reviewers' box. If you do not agree with any of their criticisms or suggestions please explain clearly why this is so.

Reviewer 1

Advance summary and potential significance to field

The function of nuclear lamina proteins is an interesting and timely topic. Beyond the mere mechanical function to stabilise the nuclear envelope and protect the chromosomes, lamina proteins are involved or are assumed to be involved an a long list of cellular and nuclear functions. The authors investigate the potential regulation of the protein BAF, which is enriched at the nuclear lamina. The authors propose that BAF localisation and function is controlled by the mechanics of muscle cells and that BAF feeds into cell cycle control in that BAF suppresses entry into endocycles. The manuscript follows a previous study from Dr Volk's laboratory, Wang et al 2018.

The claims of the manuscript are that BAF represses endoreduplication, that the lamina enrichment of BAF depends on the LINC complex, the kinase Ball and the sarcomere-nucleus connection. The authors claim that loss of BAF lamina localisation leads to an increased DNA content, increased nucleoplasmic E2F1 levels. The authors claim that the effect of BAF on DNA levels would be mediated by the increased nucleoplasmic E2F1 levels. Finally the authors propose a model that BAF lamina localisation would be a mechanosensor which controls endoreduplication.

The authors present largely histological data, analysed in fixed and stained embryos. The stainings were quantified by fluorescence levels in many nuclei of four larvae. Direct quantifications of tagged proteins (such as GFP-BAF) are not included in the manuscript. The authors show loss of BAF enrichment at the lamina in koi, klar-Msp300 mutants, sis (titan) RNAi and ball RNAi larvae. The increased nucleoplasmic E2F1 staining was detected in baf RNAi larvae. Increased DAPI staining was quantified in baf, sis, ball RNAi larvae.

Comments for the author

In my view the conclusions of the manuscript are disconnected from the data. In best case (after all technical issues were resolved), the authors demonstrate a dependance of BAF protein distribution on other lamina proteins. The data do not indicate that BAF localisation would be mechanosensitive and that BAF would respond to mechanical stimuli to control the cell cycle. The central conclusion as expressed in the title - coupling of nuclear mechanics with endoreduplication - is not related to the presented data.

The data do not demonstrate a function of BAF for endoreplication. A change of DNA content as reported (factor of less than 2) may be caused by many different mechanisms, such as G2 arrest, uncontrolled DNA replication. For an endoreduplication, it is required to clarify the cell cycle stage of the cells (G1 or G2 arrest) and that cells have restarted DNA replication after completion of a previous round of the cell cycle without mitosis (S phase and G2 phase). In the previous paper the authors have employed labelling of newly synthesised DNA. Among other assays (e. G. FUCCI, Cyclin staining,…) this would demonstrate the timing and range of DNA synthesis in the muscle cells.

In my view the authors over/misinterpret their data. Although I find the description of BAF lamina staining to a certain degree convincing (if technical issues were resolved), this does not allow to conclude that this change in localisation leads to a resulting phenotype. Other aspects of BAF dynamics or biochemical modifications may also change. In best case the authors can make a correlative statement. In summary the manuscript remains on a rather descriptive levels about factors controlling distribution of BAF. The functional claims of the manuscript are not supported by data.

Specific issues (I mention here only major issues).

© 2020. Published by The Company of Biologists under the terms of the Creative Commons Attribution License (https://creativecommons.org/licenses/by/4.0/).

The quantification procedure included a context/region specific background subtraction (rolling ball). In my view it is mandatory in quantification to conduct any procedure on an image on all pixels to the same degree.
Do the results depend on the background subtraction?

The quantification would be more convincing, if an internal standard were used. I do not understand why quantifications were not normalised by the LaminC staining. An internal standard would avoid any mutant specific changes in morphology, such as imaging depth or nuclear morphology.

The quantifications would be more convincing, it the indirect measurements of two-step immunostainings were at least complemented by direct quantification of protein distribution such as BAF-GFP (which seems to be available), which are much less prone to experimental distortions.

Fig. 4G What is the ordinate? Please label.
Fig. 6C The distribution of nucleoplasmic E2F1 staining in baf RNAi embryos is bimodal. It is not informative and misleading to calculate simply an average and use gaussian statistics in such a case.

The term „expansion microscopy" is misleading, since it is merely based on classical microscopy of extremely swollen specimen.

The figures appear partly as „draft versions". The labelling is not consistent, multiple font sizes are used some are not legible (e. g. labelling in Fig 2C, 2D). Some panels have no label (e. g. image between panels 2C and 2D).

Reviewer 2

Advance summary and potential significance to field

This interesting manuscript by the Volk lab investigates a novel mechanism how DNA endoreplication can be controlled in a post mitotic tissue, in this specific case differentiated Drosophila larval muscles. The authors find that a small protein called BAF concentrates at the nuclear envelope of larval muscles. This concentration requires an intact LINC complex and normal sarcomeric content (or at least normal Drosophila titin levels) and thus likely a mechanical coupling of the nuclei to the underlying sarcomeric cytoskeleton, which is under strong mechanical tension. BAF concentration to the nuclear membrane also requires the presence of nuclear kinase called Ball and likely a normal phosphorylation pattern of BAF. Interestingly, if the nuclear membrane localisation of BAF is disturbed then a regulator of endoreplication called E2F1 is also freed from the nuclear membrane towards the nucleoplasm where it can promote endoreplication.
In summary, these findings are interesting and novel and the experiments have been well performed and controlled.

Comments for the author

1. I wonder if the authors could address one important point, which would make this paper even stronger. If not experimentally at least in the discussion. This addresses the functional relevance of the BAF localisation and its impact on endoreplication. What is the muscle phenotype upon BAF knock-down? Endoreplication is promoted but do these muscles grow more or less? Can the larva move normally and develop into adults?
I wonder what would be the biological sense that reduced mechanical force on the nucleus promotes endoreplication? Intuitively, I would have expected the opposite: high stretch could indicate a too short muscle fiber stretched between the tendons across the segment. This fiber should grow, so rather increase than decrease DNA content.
2. The authors should move the phospho mimic (BAF-3D) and the non phosphorylatable BAF-3A experiments to the results part. These are important results that should not appear in the discussion. I wonder if these have gain of function phenotypes on E2F1 localisation and on muscle

© 2020. Published by The Company of Biologists under the terms of the Creative Commons Attribution License (https://creativecommons.org/licenses/by/4.0/).

growth or function in general. It is striking that the 3A version is highly concentrated in the nucleus but possibly not at the nuclear membrane. Does this have functional consequences?

Minor points:
1. page 4 'BAF localisation…depends on its kinase activity'; should rather read on its phosphorylation state?
2. page 23, figure legend 1: 'Lamin peaks'
3. Figures 3 and 4, y, w should be in small letters.

Reviewer 3

Advance summary and potential significance to field

This manuscript by Unnikannan et al. investigates the mechanisms that lead to an increase in nuclear DNA content in polyploid Drosophila larval muscles cells with disrupted nuclear anchorage. Previous studies by the Volk group have reported that mutations in the LINC complex disrupted myonuclear anchorage to the sarcomeres and increased DNA content. To identify effectors of the LINC complex responsible for these effects, a screen was performed (Wang et al., 2018) which lead to the identification of BAF. BAF is a known chromatin regulator and has been shown in other contexts to regulate DNA organization and transcription. By analyzing protein levels and distribution on confocal images of different mutant/knockdown muscles, the authors show that components of the LINC complex and titin a protein required to anchor the nucleus to the sarcomere, are required for the enrichment of BAF at the nuclear envelope. This enrichment requires phosphorylation of BAF by its kinase Ball, and correlates with an increase in DNA content (via DAPI fluorescence intensity). To complete a linear path from the LINC complex to DNA content, the authors show that knockdown of BAF and Ball increase nuclear levels of E2F1, a known transcriptional regulator of endoreplication, which in control nuclei is preferentially located to the nuclear envelope.
The authors propose that under normal conditions, BAF is phosphorylated by Ball and enriched at the nuclear envelope, together with E2F1. In response to mechanical destabilization via the LINC complex, BAF becomes de-phosphorylated and releases E2F1 to promote endoreplication. The problem being investigated and presented data are interesting and would help in our understanding of how the LINC complex modulates DNA organization content and transcription. This work builds on published data in the field (e.g. LINC complex involvement with mechanotransduction and nuclear stiffness via Lamin and Emerin eg Guilluy et al., 2014; LINC mutations associated with E2F and endoreplication, Wang et al., 2018). This work could represent an important connection between mechanotransduction mediated by the LINC complex and changes in DNA content/organization/transcription.

Comments for the author

Despite an exciting question and interesting results, additional analyses of the existing data need to be included to support the conclusions drawn by the authors. Further, the proposed model requires additional (e.g. rescue) experiments to confirm the indicated molecular links. In addition, the text of this manuscript needs revising. Below is a list of major general issues followed by minor comments on results/figures and discussion.

General comments:

1. BAF protein localization in control muscles:
a. In all control plots (Fig.1D, 3E), BAF intensity peaks outside the LamC (inner nuclear membrane) peak. In support of this, Figure S1 C,D shows co-localization of BAF protein with peri-nuclear microtubule network. BAF has been reported to have functions outside the nucleus (Kobayashi et al., PNAS 2015; Segura-Totten and Wilson, 2004 review). It is not clear why this pool isn't taken into consideration. Likewise Ball, its kinase appears highly expressed in the cytosol.
b. BAF localization often appears punctate in the LINC complex mutations (Fig 1B",C"). Is this just the images chosen or does it reveal some aspect of the BAF interactions with components of the nuclear envelope? Are the authors suggesting that BAF directly binds to the LINC complex? The

© 2020. Published by The Company of Biologists under the terms of the Creative Commons Attribution License (https://creativecommons.org/licenses/by/4.0/).

authors did report trying to co-ip BAF with MSP-300 and indicated that it was not successful. How then is BAF localized to the inner nuclear envelope via LINC complex activity?
c. BAF protein surrounding the nucleolus: Has this been seen in other systems? Does it change in any of the genotypes? What are the functional implications for BAF at this location?
d. Cytoplasmic BAF levels look fairly similar in all three genotypes despite "reduction of transcription in LINC mutants" (Fig1A-C). In addition, BAF protein levels in the sarcomeres seem similar in control and BAF KD muscles (FigS1). Does this reflect incomplete BAF KD or antibody artifacts? Could western analyses coupled to qRTPCR be included to support the authors' conclusions?

2. BAF function:
a.BAF de-/phosphorylation changes its interaction with LEM proteins and dsDNA, which is mediated by the kinase Ball (see introduction and Segura-Totten and Wilson, 2004).
Interactions with LEM proteins as well as with dsDNA could be contributing to the phenotypes observed in this study and can be tested using published reagents. This would strengthen the model that the authors propose.
b. Is muscle function affected in the BAFkd and overexpression experiments?
c. Are the levels of the phosphomimic and the phosphomutant BAF the same? How much overexpression is found? Does the BAF-GFP constructs rescue the BAF kd, which would suggest that the tags do not affect BAF function?
b. Does the phosphomimic form of BAF rescue the Ball kd? Does it recruit E2F to the nuclear envelope as the model would suggest? The phosphomimic version maybe best to use in the co-IP with E2F. Is knockdown of BAF or BALL rescued by a reduction of E2F?

3. Quantification of DAPI intensity/DNA content:
a. Published work has shown that in the Drosophila larvae the amount of DNA per nucleus depends on the number of nuclei per cell as well as on the size of the muscle (Windner et al. 2019). In this light it is important to confirm for each genotype, and specifically in the cells that were analyzed, that there are no differences were detected in nuclear number and cell size. Alternatively, the measured DNA content in this study should be normalized for these parameters.
b. Recent data also showed that Drosophila myonuclei can contain different amounts of DNA (16, 32 or 64 copies) within the same cell (Windner et al. 2019). Does the increase in DNA content observed in this study reflect an increased number of full endocycles? It is possible that, the endocycle program is disrupted by chromatin changes in nuclei with destabilized nucleoskeleton, which could result in partial/defective DNA endoreplication.

4. Line plots for protein localization:
a. For consistency and reproducibility, lines should be placed so that all of them go through the nuclear envelope as well as the nucleolus. The authors should also superimpose traces from multiple nuclei (with error bars) as is done in other publications for this type of line analysis.
b. For a full picture, additional panels are needed that show orthogonal views of the example nuclei and the location of z-slice selected for quantification. This will also allow the reader to appreciate the full shape of the nuclei in all genotypes.
c.In addition, it would be helpful to also include DAPI intensity in these plots, as this provides an additional landmark for determining protein distribution.

5. Quantification of fluorescence intensities:
a. To better understand how the different cellular/nuclear compartments were selected for measurements (e.g. Fig1 G-I), it would be helpful to add example images with ROIs to Figure S1.
b."Automated, unbiased quantification" — all quantification should be unbiased.

6. How do the proteins investigated in this study regulate endoreplication under normal conditions? Does localization of BAF / Ball / E2F1 change during endoreplication?
Mechanical input:
a.In addition to general cytoskeletal/mechanical stability common to all cells, muscle fibers experience additional forces during muscle contractions. This distinguishes mechanics in muscle cells from other cell types and should be included in the discussion. How/Does this play into regulating endoreplication in muscle nuclei?

© 2020. Published by The Company of Biologists under the terms of the Creative Commons Attribution License (https://creativecommons.org/licenses/by/4.0/).

b. Since no mechanical data are directly collected in this study, and changes of the mechanical environment are inferred from cytoskeletal manipulations, it would be better to move mechanical implications to the discussion.
c. In light of this, titin knockdown was used to disrupt the association between nuclei and the sarcomeres. However data from other systems suggest that there is nuclear titin which interacts with lamin and influences DNA organization (Zastrow et al., J Cell Sci 2006). This may complicate the authors' interpretation and should be clarified. Could there be additional direct test of mechanical changes on the nuclei?

7. Sample size and t-test results:
a. For each experiment, the text states "taken from 4 distinct muscles no. 7, and from 4 discrete larvae", however the numbers of analyzed nuclei range from ~60 to more than 100.
Since muscle 7 (VL4) contains on average 9 nuclei, these numbers do not make much sense.
It would be great to have the actual sample sizes (number of cells) associated with each experiment. In addition, it would be important to know which abdominal hemisegment were included in the analysis.
b. To avoid repetitions, the "n=" and "p<" statements could be shortened and/or added to the figure legends.
c. controls: The Drosophila field has moved to use, for example, Mef2Gal4>UAS-GFP RNAi for knockdown control and Mef2Gal4> UAS-GFP for the overexpression control.

8. Image color selection:
For inclusiveness, it would be great to not overlay red and green colored images, as these colors are hard to distinguish for some people. Magenta and lime green are usually used.

Results:

Figure 1
a. A,B,C: Panels with only BAF antibody are needed to better see the protein pattern. Consider showing in greyscale instead of red.
b. Are BAF levels at the nucleolus reduced in control nuclei?
c. Are Lamin or fly Emerin levels reduced in LINC complex mutants?

Figure 2
a. BAF over expression seems to cause severely disrupted nuclear shapes - is this due to effects on the LINC complex, other cytoskeletal elements or both?
b. BAF localization in 2A appears lower than Fig1B c. Overexpressed BAF does not seem to localize to any landmarks (e.g. around nucleoli) Does the construct protein localize properly in other cell types and rescue the mutant phenotype?

FigureS2:
a. Line plots for protein distribution needed.
b. How much overexpression is achieved? Western analysis?

Figure 3
a. Better BAF/nucleus volume instead of current G.

Figure 4
a. Text states "Ball labeling was specifically observed in the myonuclei". However, the pattern seems much more complex, with labeling in the cytoplasm (sarcomeric pattern?) and around the nuclei (peri-nuclear microtubules?).
b. Nuclei appear clumped in the Ball RNAi. Is this also seen in the BAF RNAi? Could this mispositioning affect DNA content?

Figure S3
a. Fig. S3 does not really show "the efficiency of the RNAi". Is the cytosolic localization of Ball increased in the RNAi?
b. Figure S3 and S4 needs full sets of line plots (protein distribution).

© 2020. Published by The Company of Biologists under the terms of the Creative Commons Attribution License (https://creativecommons.org/licenses/by/4.0/).

Figure S4
a. This would be better as part of the main Fig.
c. How does NPC labeling specially compare to LamC?

Figure 6
a. A-B indicate highly increase E2F1 intensity in the nucleoplasm upon BAF KD, while levels at the nuclear envelope and in the cytoplasm are similar. This indicates higher nuclear E2F1levels, not necessarily any location changes. Please clarify.
b. Lamin levels look increased in B' - is this a phenotype?

Figure 7
a.Images and plots indicate a change in E2F1 distribution upon Ball KD. In addition to absolute levels of E2F1 in the nuclei, it would help to see envelope vs. nucleoplasm vs cytoplasm plots (like in Fig1) to make this point.

Figure 8:
a. It would be helpful to include the nucleoli, DNA, Titin and sarcomeres in the schematic (as they were discussed) and please show all the proposed mechanisms.
b. What is the meshwork along the inner nuclear membrane? Lamin?
c. LEM domain proteins are only briefly mentioned in the discussion. Some background is required to appreciate their involvement in the proposed mechanism.
d. What role do nuclear pores play here?
e. To better understand the different genotypes used in this study it would be helpful to see where KASH domains are located.

Discussion:
a.It would be great if the following topics would be included accumulation of proteins around the nucleoli nuclear positioning phenotype in BALL KD muscles DNA content increases to different amounts under different KD conditions b.It would be good to see the data mentioned in the discussion as part of the results.
Introduction: first sentence is unclear and needs to be revised.
The paper should be carefully revised as there are many typos and awkward sentences.

First revision

Author response to reviewers' comments

Response to reviewers:

Reviewer no. 1 (major comments):
In my view the conclusions of the manuscript are disconnected from the data. In best case (after all technical issues were resolved), the authors demonstrate a dependance of BAF protein distribution on other lamina proteins.

I disagree with the reviewer. Our data demonstrating that the specific localization of BAF at the nuclear envelope depends on the LINC complex is novel and never been described. The LINC complex is not part of the nuclear lamina, it bridges between the cytoskeleton and the nuclear lamina, thereby capable of transmitting mechanical signals produced by the cytoskeleton to the nuclear lamina. Elimination of BAF from the nuclear envelope in both LINC complex mutants as well as following disruption of the nuclear-sarcomeres connections indicates a critical dependence of BAF localization at the nuclear envelope in cytoplasmic mechanical inputs.

The data do not indicate that BAF localisation would be mechanosensitive and that BAF would respond to mechanical stimuli to control the cell cycle. The central conclusion as expressed in the title - coupling of nuclear mechanics with endoreduplication - is not related to the presented data.

© 2020. Published by The Company of Biologists under the terms of the Creative Commons Attribution License (https://creativecommons.org/licenses/by/4.0/).

Our interpretation that BAF localization at the nuclear envelope is mechanosensitive is based on two observations: First, BAF dissociates from the nuclear envelope in all mutants lacking components of the LINC complex, considered by numerous studies to represent the major complex that transmits mechanical inputs from the cytoskeleton to the nucleoskeleton, thus linking BAF localization at the nuclear envelope with mechanical inputs transmitted across the nuclear envelope. Second, we show that BAF localization at the nuclear envelope is similarly abrogated following temporal dissociation of the nuclear-sarcomeres connections which promoted nuclear shape deformations from oval to spheroid shapes. Such nuclear deformations were previously observed in both the LINC complex mutants, as well as following EB1 and Shortstop knockdown, indicative of changes in the nuclear mechanical environment. This is now better explained in the Discussion (see below):

“In the Drosophila muscle fibers, we found that BAF was detected in various subcellular sites, including the cytoplasm, the nuclear envelope, the nucleoplasm and at the nucleolus borders. Yet, only the portion of BAF localized at the nuclear envelope was found to change following elimination of a functional LINC complex. Since the LINC complex transmits cytoplasmic mechanical inputs from the cytoskeleton to the nucleoskeleton in various cell types (Chambliss et al., 2013) (Guilluy et al., 2014), and based on the notion that nuclear deformations (from oval into spheroid shape) was observed in both larval muscles of LINC complex mutants (Elhanany-Tamir et al., 2012), as well as in conditions where nuclei partially detached from the sarcomeres following either knockdown of EB1 and Shortstop (Wang et al., 2015), or Sls (shown here), it is suggestive that nuclear deformations are indicative of altered mechanical inputs applied on the nuclear envelope. It is therefore concluded that maintenance of BAF at the nuclear envelope is mechanically sensitive”.

The data do not demonstrate a function of BAF for endoreplication. A change of DNA content as reported (factor of less than 2) may be caused by many different mechanisms, such as G2 arrest, uncontrolled DNA replication. For an endoreduplication, it is required to clarify the cell cycle stage of the cells (G1 or G2 arrest) and that cells have restarted DNA replication after completion of a previous round of the cell cycle without mitosis (S phase and G2 phase). In the previous paper the authors have employed labelling of newly synthesised DNA. Among other assays (e. G. FUCCI, Cyclin staining,…) this would demonstrate the timing and range of DNA synthesis in the muscle cells.

Many studies indicated that Drosophila muscle nuclei undergo endoreplication (e.g. Windner et al., 2019). Our previous report (Wang et al, 2018), demonstrated that increased endoreplication (denoted by EdU incorporation into the DNA of larval muscles nuclei) was observed in all the LINC mutants. This resulted with elevated levels of DNA (measured by DAPI integrated density) in all muscle nuclei. Based on this, in the present manuscript we only measured DAPI integrated density to assess the extent of DNA endoreplication and indeed demonstrate that in the various experimental setups (BAF KD, Ball KD, and sls temporal KD) the level of DNA endoreplication increased.

In my view the authors over/misinterpret their data. Although I find the description of BAF lamina staining to a certain degree convincing (if technical issues were resolved), this does not allow to conclude that this change in localisation leads to a resulting phenotype. Other aspects of BAF dynamics or biochemical modifications may also change. In best case the authors can make a correlative statement. In summary the manuscript remains on a rather descriptive levels about factors controlling distribution of BAF. The functional claims of the manuscript are not supported by data.

To the best of my knowledge, this is the first time to show that E2F1, a key regulator of endoreplication is localized at the nuclear envelope. This provides a novel aspect of cell cycle control by factors localized at the nuclear envelope. Release of E2F1 from the nuclear envelope into the nucleoplasm might increase the extent of endoreplication, assuming that its levels are limiting. Our data indicate that both BAF and E2F1 colocalize at the nuclear envelope, and that loss of BAF from the nuclear envelope (which appears to be mechano-sensitive as explained above), correlated with a loss of E2F1 from the nuclear envelope, and a concomitant increase of its levels in the nucleoplasm. Although the precise mechanism of E2F1 release from the nuclear envelope into the nucleoplasm is currently unclear our experiments suggest a novel model by which E2F1 localization at the nuclear envelope, is similarly to BAF depends on mechanosensitive signals applied on the nuclear envelope.

© 2020. Published by The Company of Biologists under the terms of the Creative Commons Attribution License (https://creativecommons.org/licenses/by/4.0/).

Specific issues raised by this reviewer:
The quantification procedure included a context/region specific background subtraction (rolling ball). In my view it is mandatory in quantification to conduct any procedure on an image on all pixels to the same degree. Do the results depend on the background subtraction?

Rolling ball is a conventional method for background subtraction. However, the results were clear even without applying this method. In the course of our experimental routine, we add the antibody mix to all larvae at the same tube (labeling of larvae for each genotype is done by cutting the heads, tails, or both), and the numbers are presented. Furthermore, each of the experiments presented in the manuscript was repeated 3 times and the results indicated a similar trend (although the numbers of integrated fluorescence density differed).

The quantification would be more convincing, if an internal standard were used. I do not understand why quantifications were not normalised by the LaminC staining. An internal standard would avoid any mutant specific changes in morphology, such as imaging depth or nuclear morphology.

Since lamin C levels change under the different genotypes, normalization to lamin C levels would be misleading.

The quantifications would be more convincing, it the indirect measurements of two-step immunostainings were at least complemented by direct quantification of protein distribution such as BAF-GFP (which seems to be available), which are much less prone to experimental distortions.

Overexpression of BAF-GFP would not necessarily recapitulate BAF endogenous levels. Furthermore, we show that BAF-GFP does not recapitulates 100% the endogenous localization of BAF observed with antibody staining.

Fig. 4G What is the ordinate? Please label.

Done.

Fig. 6C The distribution of nucleoplasmic E2F1 staining in baf RNAi embryos is bimodal. It is not informative and misleading to calculate simply an average and use gaussian statistics in such a case.

The distribution of BAF was quantified only at the nuclear envelope and not around the nucleolus, thus it is not bimodal.

The term „expansion microscopy“ is misleading, since it is merely based on classical microscopy of extremely swollen specimen.

The word “Expansion microscopy” was termed by Chen et al., Science. 347:543-548.

The figures appear partly as „draft versions“. The labelling is not consistent, multiple font sizes are used, some are not legible (e. g. labelling in Fig 2C, 2D). Some panels have no label (e. g. image between panels 2C and 2D).

All fonts were correctly inserted.

Reviewer no 2:

1. I wonder if the authors could address one important point, which would make this paper even stronger. If not experimentally at least in the discussion. This addresses the functional relevance of the BAF localisation and its impact on endoreplication. What is the muscle phenotype upon BAF knock-down? Endoreplication is promoted but do these muscles grow more or less? Can the larva move normally and develop into adults?

© 2020. Published by The Company of Biologists under the terms of the Creative Commons Attribution License (https://creativecommons.org/licenses/by/4.0/).

The functional relevance of increased endoreplication is an important question. Since BAF mutants do not survive to the larval stage we were unable to test this question in muscles that lack completely BAF function. Since the knockdown of BAF in muscles is partial it is difficult to estimate the full contribution of BAF to muscle growth and function. The baf KD larvae described in our experiments did not show overgrowth of the muscles, and developed into adult flies with no obvious muscle phenotype. We added a sentence in the Discussion (page 13) that explains this: "From physiological point of view, there no detectable change in muscle size, or movement was observed in the baf KD muscles, and the larvae developed up to adult stage. baf homozygous mutant did not develop up to the 3rd instar larval stage, so the full physiological contribution of BAF to muscle growth awaits experiments in which a more efficient reduction of BAF levels in the muscle tissue will be induced."

I wonder what would be the biological sense that reduced mechanical force on the nucleus promotes endoreplication? Intuitively, I would have expected the opposite: high stretch could indicate a too short muscle fiber stretched between the tendons across the segment. This fiber should grow, so rather increase than decrease DNA content.

This is an interesting question. Whether the mechanical force on the nuclear envelope increases, or decreases in the LINC mutants, or following dissociation of nuclei from sarcomeres is unclear at this stage. Our previous studies (Wang et al., 2018 and Lorber et al., 2020) indicated that the LINC complex might be required for equalizing the mechanical forces applied on the nuclear envelope of the multiple nuclei in a given muscle fiber. It is therefore possible that mechanical forces on the nuclear envelope at this stage control synchronization of activities (including endoreplication) between all the muscle nuclei of a given fiber.

2. The authors should move the phospho mimic (BAF-3D) and the non phosphorylatable BAF-3A experiments to the results part. These are important results that should not appear in the discussion. I wonder if these have gain of function phenotypes on E2F1 localisation and on muscle growth or function in general. It is striking that the 3A version is highly concentrated in the nucleus but possibly not at the nuclear membrane. Does this have functional consequences?

As suggested by the reviewer we transferred the results of overexpression of the various BAF-GFP constructs from supplement to the results section and now are included in new Figure 6. The expression of BAF-3A led to a dominant effect on muscle fibers integrity, and these larvae did not developed into adult flies. Based on the ability of BAF-3A to bind avidly dsDNA, and the observation that DNA distribution appeared abnormal in the nuclei overexpressing BAF-3A, we speculate that transcription is abnormal in these muscles, and consequently this led to functional defects in the larval muscle fibers.

Minor points:
1. page 4 'BAF localisation…depends on its kinase activity'; should rather read on its phosphorylation state?
Thanks, corrected.

2. page 23, figure legend 1: 'Lamin peaks'
Thanks, corrected.

3. Figures 3 and 4, y, w should be in small letters.
Thanks, corrected.

Reviewer no 3:
Despite an exciting question and interesting results, additional analyses of the existing data need to be included to support the conclusions drawn by the authors. Further, the proposed model requires additional (e.g. rescue) experiments to confirm the indicated molecular links. In addition, the text of this manuscript needs revising. Below is a list of major general issues, followed by minor comments on results/figures and discussion.

Thanks for the positive view of the manuscript. Regarding rescue experiments, baf mutants are not viable at the 3rd instar larval stage, thus rescue experiments with muscle-driven BAF cannot be performed.

© 2020. Published by The Company of Biologists under the terms of the Creative Commons Attribution License (https://creativecommons.org/licenses/by/4.0/).

General comments:

1. BAF protein localization in control muscles:
a. In all control plots (Fig.1D, 3E), BAF intensity peaks outside the LamC (inner nuclear membrane) peak. In support of this, Figure S1 C,D shows co-localization of BAF protein with peri-nuclear microtubule network. BAF has been reported to have functions outside the nucleus (Kobayashi et al., PNAS 2015; Segura-Totten and Wilson, 2004 review). It is not clear why this pool isn't taken into consideration. Likewise Ball, its kinase appears highly expressed in the cytosol.

Although we show that BAF localizes in the cytoplasm, and partially overlaps the microtubule cytoskeleton, the major effect on BAF distribution observed in the LINC mutants, ball KD, and sls KD muscles is its deficiency at the nuclear envelope. The entire manuscript focus on BAF localization at the nuclear envelope, and all quantifications were performed accordingly.

b. BAF localization often appears punctate in the LINC complex mutations (Fig 1B",C"). Is this just the images chosen or does it reveal some aspect of the BAF interactions with components of the nuclear envelope? Are the authors suggesting that BAF directly binds to the LINC complex? The authors did report trying to co-ip BAF with MSP-300 and indicated that it was not successful. How then is BAF localized to the inner nuclear envelope via LINC complex activity?

Based on the literature BAF might bind to one or more representatives of the LEM domain proteins family. We now show that the LEM protein Otefin significantly reduced from the nuclear envelope in all LINC mutant muscles (new Figure 3). Therefore, a plausible scenario is that the LINC complex maintains the localization of Otefin at the nuclear envelope, and phosphorylated BAF binds directly to Otefin. The absence of Otefin from the nuclear envelope leads to dissociation of BAF from the nuclear envelope. This is now explained in the Discussion and in the revised model.

c. BAF protein surrounding the nucleolus: Has this been seen in other systems? Does it change in any of the genotypes? What are the functional implications for BAF at this location?

We do not know whether BAF has a function in determination of the nucleolus borders, however we feel this aspect is not directly related to the present study.

d. Cytoplasmic BAF levels look fairly similar in all three genotypes despite "reduction of transcription in LINC mutants" (Fig1A-C). In addition, BAF protein levels in the sarcomeres seem similar in control and BAF KD muscles (FigS1). Does this reflect incomplete BAF KD or antibody artifacts? Could western analyses coupled to qRTPCR be included to support the authors' conclusions?

A major point of our manuscript is that despite an overall reduction of BAF mRNA reported in our previous study (Wang et al 2018), the key site in which BAF protein changes its distribution is the nuclear envelope. Western analysis does not work with the anti BAF antibodies, and in any case it would not indicate the specific decrease in BAF protein at the nuclear envelope.

2. BAF function:
a.BAF de-/phosphorylation changes its interaction with LEM proteins and dsDNA, which is mediated by the kinase Ball (see introduction and Segura-Totten and Wilson, 2004).
Interactions with LEM proteins as well as with dsDNA could be contributing to the phenotypes observed in this study and can be tested using published reagents. This would strengthen the model that the authors propose.

As pointed above (Rev # 2) we show that Otefin levels at the nuclear envelope decrease significantly in all LINC mutants muscles (new Figure 3). We also added the results of overexpression of the BAF phospho-mutants to indicate the contribution of the phosphorylation state of BAF to its localization at the nuclear envelope (new Figure 6).

b. Is muscle function affected in the BAFkd and overexpression experiments?
Our measurements did not indicate a change in muscle size, larval locomotion, or adult fly hatching. This is indicated in the Discussion session: "From physiological point of view, there no

© 2020. Published by The Company of Biologists under the terms of the Creative Commons Attribution License (https://creativecommons.org/licenses/by/4.0/).

detectable change in muscle size, or movement was observed in the baf KD muscles, and the larvae developed up to adult stage. baf homozygous mutant did not develop up to the 3rd instar larval stage, so the full physiological contribution of BAF to muscle growth awaits experiments in which a more efficient reduction of BAF levels in the muscle tissue will be induced.”

c. Are the levels of the phosphomimic and the phosphomutant BAF the same? How much overexpression is found? Does the BAF-GFP constructs rescue the BAF kd, which would suggest that the tags do not affect BAF function?

Although we used the same mef2-Gal4 driver to drive each of the BAF-GFP constructs, we find that the levels of the various BAF-GFP proteins differed. This was concluded from Western analysis of larval extracts using anti GFP antibodies. Furthermore, the GFP-BAF-3D construct did not recapitulate the expected localization at the nuclear envelope, and was rather spread all over the muscle cytoplasm. Therefore we did not perform additional experiments with it. As explained above, rescue experiments cannot be performed since the baf mutants do not survive up to 3rd instar larvae stage.

b. Does the phosphomimic form of BAF rescue the Ball kd? Does it recruit E2F to the nuclear envelope as the model would suggest? The phosphomimic version maybe best to use in the co-IP with E2F. Is knockdown of BAF or BALL rescued by a reduction of E2F?

We tried to rescue the koi/Sun mutant muscles by overexpression of the GFP-BAF-3D (phosphomimic BAF) construct, however we did not get rescue of E2F1 at the nuclear envelope. Since the GFP-BAF-3D protein did not recapitulate the localization of BAF at the nuclear membrane, we think this result is not conclusive and therefore we did not add it to the manuscript.

3. Quantification of DAPI intensity/DNA content:
a. Published work has shown that in the Drosophila larvae the amount of DNA per nucleus depends on the number of nuclei per cell as well as on the size of the muscle (Windner et al. 2019). In this light it is important to confirm for each genotype, and specifically in the cells that were analyzed, that there are no differences were detected in nuclear number and cell size. Alternatively, the measured DNA content in this study should be normalized for these parameters.

In all mutants analyzed the number of nuclei per muscle did not change. We do find size differences between the larvae with aberrant muscle organization (e.g. sls KD muscles, or following overexpression of BAF -3A). However, in these instances the amount of DNA in the muscle nuclei increased despite of the smaller fiber size.

b. Recent data also showed that Drosophila myonuclei can contain different amounts of DNA (16, 32 or 64 copies) within the same cell (Windner et al. 2019). Does the increase in DNA content observed in this study reflect an increased number of full endocycles? It is possible that, the endocycle program is disrupted by chromatin changes in nuclei with destabilized nucleoskeleton, which could result in partial/defective DNA endoreplication.

Our measurements do not support 16, 32 or 64 copies as described in Winder et al., 2019.

4. Line plots for protein localization:
a. For consistency and reproducibility, lines should be placed so that all of them go through the nuclear envelope as well as the nucleolus. The authors should also superimpose traces from multiple nuclei (with error bars) as is done in other publications for this type of line analysis.

The line plots in our measurements the line plots go always through the nuclear center. The nucleolus does not localize always at the nuclear center. We do have error bars in the box plots.

b. For a full picture, additional panels are needed that show orthogonal views of the example nuclei and the location of z-slice selected for quantification. This will also allow the reader to appreciate the full shape of the nuclei in all genotypes.

© 2020. Published by The Company of Biologists under the terms of the Creative Commons Attribution License (https://creativecommons.org/licenses/by/4.0/).

Since the figures are rather full with multiple panels and orthogonal view does not add essential information to the panels, I feel this is unnecessary. Orthogonal view was added to Figure 4 (former Fig 3) to illustrate nuclear deformations at the Z axis.

c.In addition, it would be helpful to also include DAPI intensity in these plots, as this provides an additional landmark for determining protein distribution.

DAPI staining is presented in all Figure panels. For the intensity profile, I do not think it will add informative data.

5. Quantification of fluorescence intensities:
a. To better understand how the different cellular/nuclear compartments were selected for measurements (e.g. Fig1 G-I), it would be helpful to add example images with ROIs to Figure S1.

The quantification procedure is detailed in the “Data Collection” under Materials and Methods section. The macro has been deposited in GitHub DOI 10.5281/zenodo.3372266.

b.“Automated, unbiased quantification” — all quantification should be unbiased.

I agree. The sentence was corrected.

6. How do the proteins investigated in this study regulate endoreplication under normal conditions? Does localization of BAF / Ball / E2F1 change during endoreplication?

We suggest that proteins at the nuclear envelope tether E2F1 at the nuclear envelope, thus controlling its release into the nucleoplasm. To address changes in BAF / Ball / E2F1 during endoreplication we will have to follow their levels in live larvae for long periods of time because endoreplication takes place throughout larval stages. Unfortunately, we do not have currently such reagents.

Mechanical input:
a.In addition to general cytoskeletal/mechanical stability common to all cells, muscle fibers experience additional forces during muscle contractions. This distinguishes mechanics in muscle cells from other cell types and should be included in the discussion. How/Does this play into regulating endoreplication in muscle nuclei?

This point is mentioned in the Discussion: “Coupling between endoreplication process and the mechanical environment of the cell allows the nucleus to respond to mechanical changes following tissue injury without a need for cell division. This is especially true in myofibers, where reiterated mechanical inputs are applied on the nuclear envelope during muscle contractile waves.”

b. Since no mechanical data are directly collected in this study, and changes of the mechanical environment are inferred from cytoskeletal manipulations, it would be better to move mechanical implications to the discussion.

I believe the mechanical connection is important. As discussed above we do interpret the deformation of the nuclei as a direct sign for altered mechanical environment of the nuclear envelope. Recent published data from our lab indicated that the nuclei in the LINC mutant muscles are exposed to differential mechanical environment relative to control nuclei (Lorber et al., 2020).

c. In light of this, titin knockdown was used to disrupt the association between nuclei and the sarcomeres. However data from other systems suggest that there is nuclear titin which interacts with lamin and influences DNA organization (Zastrow et al., J Cell Sci 2006). This may complicate the authors’ interpretation and should be clarified. Could there be additional direct test of mechanical changes on the nuclei?

Staining with anti Sls (Titin) antibody did not indicate nuclear staining.

7. Sample size and t-test results:

© 2020. Published by The Company of Biologists under the terms of the Creative Commons Attribution License (https://creativecommons.org/licenses/by/4.0/).

a. For each experiment, the text states “taken from 4 distinct muscles no. 7, and from 4 discrete larvae”, however the numbers of analyzed nuclei range from ~60 to more than 100.
Since muscle 7 (VL4) contains on average 9 nuclei, these numbers do not make much sense.
It would be great to have the actual sample sizes (number of cells) associated with each experiment. In addition, it would be important to know which abdominal hemisegment were included in the analysis.

In each experiment, we take in parallel 4-5 larvae of control, and 4-5 larvae of each of the mutants. The larvae were staged, and further grow in parallel up to 3rd instar larvae stage. After fixation the larvae are stained together at the same tube (each genotype is marked by physical cutting of the head, tail, or both) so that all larvae are exposed to equal antibody dilutions. Imaging is done on the same day for all samples. Muscle no. 7 from abdominal segments 2-4 of each larvae are routinely imaged and analyzed. The number of nuclei from all muscles analyzed is included. Each experiment is repeated at least 3 times, but the results shown in the graphs were taken from a single experiment.

b. To avoid repetitions, the “n=“ and “p<“ statements could be shortened and/or added to the figure legends.

We deleted information on the number of nuclei from the Figure legends, and left it in the Results section.

c. controls: The Drosophila field has moved to use, for example, Mef2Gal4>UAS-GFP RNAi for knockdown control and Mef2Gal4> UAS-GFP for the overexpression control.

We agree, but sometime it makes the sentences very confusing.

8. Image color selection:
For inclusiveness, it would be great to not overlay red and green colored images, as these colors are hard to distinguish for some people. Magenta and lime green are usually used.

This will be relevant for our next manuscript.

More points:
Figure 1
a. A,B,C: Panels with only BAF antibody are needed to better see the protein pattern. Consider showing in greyscale instead of red.
b. Are BAF levels at the nucleolus reduced in control nuclei?
c. Are Lamin or fly Emerin levels reduced in LINC complex mutants?

a. The pattern of BAF is complicated because it is observed both at the nuclear envelope as well as along the microtubules. We do not believe black/white images will change it.
b. BAF levels do not change in control muscles.
c. Lamin levels change slightly in the LINC mutants (Wang et al., 2015). We do not have antibodies to Emerin, however we show that Otefin levels decrease.

Figure 2
a. BAF over expression seems to cause severely disrupted nuclear shapes - is this due to effects on the LINC complex, other cytoskeletal elements or both?
This was not analyzed.
b. BAF localization in 2A appears lower than Fig1B
Since each experiment is performed in a separated manner, the background levels might differ. It is therefore important to compare the fluorescent levels per a given experiment in which control and mutant samples are grouped together and exposed to similar antibody dilutions.

c. Overexpressed BAF does not seem to localize to any landmarks (e.g. around nucleoli) Does the construct protein localize properly in other cell types and rescue the mutant phenotype?

It is possible that BAF overexpression does not fully recapitulate the endogenous distribution.

© 2020. Published by The Company of Biologists under the terms of the Creative Commons Attribution License (https://creativecommons.org/licenses/by/4.0/).

FigureS2:
a. Line plots for protein distribution needed.
The 2D shows that BAF localization at the nuclear membrane does not differ from that of control.

b. How much overexpression is achieved? Western analysis?
As mentioned above the Western blot with anti BAF did not reveal a clear band.

Figure 3
a. Better BAF/nucleus volume instead of current G.
We disagree. The measure indicates BAF specifically at the nuclear envelope.

Figure 4
a. Text states “Ball labeling was specifically observed in the myonuclei”. However, the pattern seems much more complex, with labeling in the cytoplasm (sarcomeric pattern?) and around the nuclei (peri-nuclear microtubules?).
We corrected the sentence to say that Ball was found to localize within the nuclei as well as along the sarcomeres. It was not found surrounding the nuclei.

b. Nuclei appear clumped in the Ball RNAi. Is this also seen in the BAF RNAi? Could this mispositioning affect DNA content?
In BAF RNAi the nuclei do not collapse. Our analysis do not show correlation between the extent of nuclear position defects and DNA endoreplication.

Figure S3
a. Fig. S3 does not really show “the efficiency of the RNAi”. Is the cytosolic localization of Ball increased in the RNAi?
No.

b. Figure S3 and S4 needs full sets of line plots (protein distribution).
Figure S4 was transferred to the results section (currently Fig 6). The line plots were added.

Figure S4
a. This would be better as part of the main Fig.
Done.
c. How does NPC labeling specially compare to LamC?
NPC labeling is punctuated rather than continuous as Lamin C.

Figure 6
a. A-B indicate highly increase E2F1 intensity in the nucleoplasm upon BAF KD, while levels at the nuclear envelope and in the cytoplasm are similar. This indicates higher nuclear E2F1levels, not necessarily any location changes. Please clarify.

It was difficult to distinguish between E2F1 levels at the nuclear envelope versus the nucleoplasm. Therefore quantification was performed on the entire nucleus including both the nuclear envelope and the nucleoplasm.

b. Lamin levels look increased in B’ - is this a phenotype?
No.

Figure 7
a. Images and plots indicate a change in E2F1 distribution upon Ball KD. In addition to absolute levels of E2F1 in the nuclei, it would help to see envelope vs. nucleoplasm vs cytoplasm plots (like in Fig1) to make this point.
As stated above, it was difficult to distinguish between E2F1 levels at the nuclear envelope versus the nucleoplasm. Therefore quantification was performed on the entire nucleus including both the nuclear envelope and the nucleoplasm.

Figure 8:
a. It would be helpful to include the nucleoli, DNA, Titin and sarcomeres in the schematic (as they were discussed) and please show all the proposed mechanisms.

© 2020. Published by The Company of Biologists under the terms of the Creative Commons Attribution License (https://creativecommons.org/licenses/by/4.0/).

We find such addition might confuse the reader.

b. What is the meshwork along the inner nuclear membrane? Lamin?
Yes.

c. LEM domain proteins are only briefly mentioned in the discussion. Some background is required to appreciate their involvement in the proposed mechanism.
This was now added in both the results section as well as in the Discussion.

d. What role do nuclear pores play here?
We do not know. They were included for understanding the context.

e. To better understand the different genotypes used in this study it would be helpful to see where KASH domains are located.
This is now described in the legends to the Model.

Discussion:
a.It would be great if the following topics would be included accumulation of proteins around the nucleoli While agreeing that accumulation of BAF around the nucleoli is interesting, the functional information for that is limiting, and we find it is not directly related to the main focus of the manuscript. nuclear positioning phenotype in BALL KD muscles
The reason for this phenotype is currently unclear, so we did not want to elaborate on this further.
DNA content increases to different amounts under different KD conditions
The reason for that could be possibly variations between larvae.

b.It would be good to see the data mentioned in the discussion as part of the results.
As mentioned above we transferred the data on BAF phosphorylation to the Results section.

Introduction: first sentence is unclear and needs to be revised.
The sentence was revised.

The paper should be carefully revised as there are many typos and awkward sentences.
OK.

Second decision letter

MS ID#: DEVELOP/2020/191304

MS TITLE: Recruitment of BAF to the nuclear envelope couples nuclear mechanics with endoreplication

AUTHORS: C.P. Unnikannan, Adriana Reuveny, Dvorah Grunberg, and Talila Volk

I have now received all the referees reports on the above manuscript, and have reached a decision. The referees' comments are appended below, or you can access them online: please go to BenchPress and click on the 'Manuscripts with Decisions' queue in the Author Area.

The overall evaluation is positive and we would like to publish a revised manuscript in Development, provided that the referees' comments can be satisfactorily addressed. In my opinion all these comments can be addressed by suitably amending the text, providing relevant clarificatio, toning down the claim that the effect observed is purel mechano-hcemical when, as several reviewers point out, the effect could be structural. Please attend to the reviewers' comments in your revised manuscript and detail them in your point-by-point response. If you do not agree with any of their criticisms or suggestions explain clearly why this is so.

© 2020. Published by The Company of Biologists under the terms of the Creative Commons Attribution License (https://creativecommons.org/licenses/by/4.0/).

We are aware that you may currently be unable to access the lab to undertake experimental revisions. If it would be helpful, we encourage you to contact us to discuss your revision in greater detail. Please send us a point-by-point response indicating where you are able to address concerns raised (either experimentally or by changes to the text) and where you will not be able to do so within the normal timeframe of a revision. We will then provide further guidance. Please also note that we are happy to extend revision timeframes as necessary.

Reviewer 1

Advance summary and potential significance to field

.

Comments for the author

The central problems with the manuscript remain. The conclusions as stated in the title and the abstract are not supported by the presented data.

1. BAF localisation. The authors show that BAF enrichment at the nuclear lamina, among other proteins of the nuclear lamina, depends on the line complex. The authors conclude that the loss of BAF enrichment in koi/klar/MSP mutants reflects a mechanism-dependent localization, because among its manifold functions the linc complex has been implicated in mechanotransduction at the nuclear envelope. The authors do not consider that the dependance of BAF enrichment is due to a structural function of the LINC complex unrelated to mechanotransduction.

The authors try to address the mechanodepandance of BAF enrichment by analysing titin RNAi depleted tissue. The authors do not consider the option that titin depletion leads to a structural change via the LINC complex. In my view, the authors show a functional dependance of BAF lamina enrichment on LINC and titin. I do not see any data that would exclude a structural dependance or point to a dependance on the nuclear mechanics.

The localization of BAF at the lamina represents an enrichment but lamina localization is certainly not exclusive. A major fraction of BAF is not localised at the lamina and appears not to depend on the linc complex. The authors focus on the fractions of BAF at the lamina. Why do the authors believe that the lamina fraction of BAF is the functionally important fraction? No data supporting this hypothesis are presented.

2. BAF function in endoreplication. I think a problem here is the definition of endoreplication. I my view endoreplication is the process of successive rounds of DNA replication without mitosis. This leads with each round to a doubling of the DNA content. The authors observe an increase but not doubling of DNA content.
Other processes than endoreplication such as uncontrolled DNA replication or locus specific DNA amplification may also lead to increased DNA content. The authors do no consider these other options. The authors do not assay other markers for cell cycle progression (FUCCI, cyclins, for example).

3. E2F1. The authors observe a dependance of E2F1 enrichment at lamina on BAF. The authors assume that changes in this enrichment reflects function. The authors do not consider that the major part of E2F1 molecules are unaffected by BAF. Why do the authors believe that only the fraction of E2F1 at the nuclear envelope would be the functionally important fraction? The authors do not present any data supporting this hypothesis.

4. technical issues
The quantification would be more convincing, if an internal standard were used. I do not understand why quantifications were not normalised by the LaminC staining. An internal standard would avoid any mutant specific changes in morphology, such as imaging depth or nuclear morphology. It is interesting to read that laminC behaves similar to BAF. This supports the idea that the LINC dependance of BAF is due to structural changes but not mechano-dependent. There are

© 2020. Published by The Company of Biologists under the terms of the Creative Commons Attribution License (https://creativecommons.org/licenses/by/4.0/).

many more lamina proteins that can be employed as internal standards for proper quantifications such as Lamin Dm0, nuclear pore proteins for example.

Fig. 8C (previous version 6C) The distribution of nuclear E2F1/nuclear volume is bimodal. The symmetrical distribution in wild type splits into two population after baf depletion. one population has a lower intensity. the second population has a higher intensity. This indicates that there are two groups of the nuclei. It is not informative to put both populations together for such a bimodal distribution. The presented bimodal distribution has nothing to do with the nucleolar localizaition.

expansion microscopy. I know that there has been a previous publication which introduced the term. Despite this the term expansion microscopy is misleading and questionable.

The quantification procedure included a context/region specific background subtraction (rolling ball). In my view it is mandatory in quantification to conduct any procedure on an image on all pixels to the same degree. As similar results are obtained without the context specific image processing (rolling ball background subtraction), I am surprised that the authors still present data which are derived from a quantification procedure containing context specific image processing.

Reviewer 2

Advance summary and potential significance to field

This revised manuscript investigates the role of BAF during larval muscle development. It finds that BAF is localized at the nuclear membrane. This localization depends on the LINC complex, on intact sarcomere organization and the kinase Ball.
The authors find that reducing BAF, ball or the sarcomeric organization results in higher DNA content in the larvae, suggesting elevated endo-replication.
Interestingly, the authors find a molecular link to a regulator of endo-replication, E2F, whose correct nuclear levels depend on Ball and BAF function.
The authors hypothesis that nuclear mechanics controls endo-replication, which needs to be regulated during larval growth.

As indicated in my initial review, I found these findings interesting, despite the missing characterization of the functional consequences after BAF knock-down.
Now, the authors added to the discussion that BAF knock-down larvae develop normally.

Comments for the author

1. The result that BAF knock-down larvae grow to adulthood is an important result which should be document in the paper in the results part. Did the authors measure growth rate and developmental time? Of course, knock-down levels can be one issue, however Baf knock-down is strong enough to cause the documented protein localization (E2F) and endo-replication defects. Further, the gain of function phenotype (BAF3D) could be explored.
If growth rate is normal in all cases, the functional link of the observed localization phenotypes in the BAF knock-down larvae, including the endo-replication defects, to larval growth is not established. This should then be clearly stated in the paper.

2. The authors again introduce new data in the discussion as unpublished results.
If these data are important for the paper, they should be added to the results section and shown. If they are not important, they should not be discussed as data not shown.

Reviewer 3

Advance summary and potential significance to field

This is a revised manuscript from Unnikannan et al. In previous work, the Volk lab has found an increase in DNA content in mutations in the LINC complex. In this work they provide data to support a linear pathway from the LINC complex to E2F1 a key regulator of DNA endoreplication. This

© 2020. Published by The Company of Biologists under the terms of the Creative Commons Attribution License (https://creativecommons.org/licenses/by/4.0/).

pathway consists of localization of BAF via its phosphorylation status and Otefin at the inner nuclear envelope. This in turn regulates E2F1 localization at the nuclear envelope. Disruption of the Linc complex or its connection to the sarcomere, leads to release of BAF (and E2F1) from the nuclear envelope into the nucleoplasm where it leads to DNA endoreplication.

Comments for the author

In this revised version, the authors present a clearer set of data and a streamlined message, in which they implicate the LINC complex in the localization of BAF at the nuclear envelope which in turn localizes, in some way, E2F1. E2F1 in turn regulates endoreplication. This mechanism explains the change in DNA content shown in the LINC mutations.
However there remain some concerns with the data as presented:

1. Based on published literature in other systems, the authors assume that in the Drosophila larval muscle system, that the LINC complex functions as a mechanotransducer. They interpret altered nuclear shape in the LINC mutants as “altered mechanotranduction”. However, the authors offer no direct measure of this. There can be other explanations for the altered shapes which have not been ruled out. Hence the “nuclear mechanics” connection in the title and in the results should be removed. The authors are free to speculate about mechanics in the Discussion section.

2. Similarly with the Otefin data, the authors assume that, since BAF has been shown in other systems to bind to Otefin, it would do the same here. No data has been shown to support this. In addition to a co-ip, the authors could show a change in Baf nuclear envelope localization in an Otefin mutant/knockdown. And what about the other LEM domain proteins? Why are they ruled out? In Otefin mutants, is there increased DNA content? Is E2F similarly mislocalized as would be predicted? The model is not fully tested.

Other issues:
The GFP-BAF, GFP-BAF-3A, GFP-BAF3D – all the levels are different in the images.
Could this influence the expression localization that is reported for each? Do E2F1 localization and DNA content change in these backgrounds as a test of the authors’ model?

BAF total levels are reduced in the SLS RNAi, could that contribute to reduced BAF localization at the nuclear envelope?

Baf reduction appears to have a greater impact on the DNA content than Ball KD or sls KD. How does this compare to LINC kd? How are E2F1 levels altered in these situations as a further test of the model?

The authors should show the remaining Baf localization in the BAF kd .. are all the levels reduced or more so at the nuclear envelope? The authors should also clarify their comment in the discussion line 298-300 “These findings are consistent with a dynamic exchange of BAF between cytoplasmic and nuclear pools where BAF in the cytoplasm primarily responds to mechanical signals” . This appears at odds with what the authors are proposing in their model.

Second revision

<u>Author response to reviewers' comments</u>

Reviewer 1 Advance summary and potential significance to field
.

Reviewer 1 Comments for the author
The central problems with the manuscript remain. The conclusions as stated in the title and the abstract are not supported by the presented data.

© 2020. Published by The Company of Biologists under the terms of the Creative Commons Attribution License (https://creativecommons.org/licenses/by/4.0/).

1. BAF localisation. The authors show that BAF enrichment at the nuclear lamina, among other proteins of the nuclear lamina, depends on the line complex. The authors conclude that the loss of BAF enrichment in koi/klar/MSP mutants reflects a mechanism-dependent localization, because among its manifold functions, the linc complex has been implicated in mechanotransduction at the nuclear envelope. The authors do not consider that the dependance of BAF enrichment is due to a structural function of the LINC complex unrelated to mechanotransduction.

The authors try to address the mechanodepandance of BAF enrichment by analysing titin RNAi depleted tissue. The authors do not consider the option that titin depletion leads to a structural change via the LINC complex. In my view, the authors show a functional dependance of BAF lamina enrichment on LINC and titin. I do not see any data that would exclude a structural dependance or point to a dependance on the nuclear mechanics.

The localization of BAF at the lamina represents an enrichment but lamina localization is certainly not exclusive. A major fraction of BAF is not localised at the lamina and appears not to depend on the linc complex. The authors focus on the fractions of BAF at the lamina. Why do the authors believe that the lamina fraction of BAF is the functionally important fraction? No data supporting this hypothesis are presented.

The claim that BAF localization at the nuclear envelope is mechanosensitive was replaced by a statement that it is LINC-and/or Titin- dependent. This was indicated in the title, as well as throughout the text (see lines 14, 89, 148, 257, 292, 317, 340, 341).

2. BAF function in endoreplication. I think a problem here is the definition of endoreplication. I my view endoreplication is the process of successive rounds of DNA replication without mitosis. This leads with each round to a doubling of the DNA content. The authors observe an increase but not doubling of DNA content.
Other processes than endoreplication such as uncontrolled DNA replication or locus specific DNA amplification may also lead to increased DNA content. The authors do no consider these other options. The authors do not assay other markers for cell cycle progression (FUCCI, cyclins, for example).

Our previous publication (Wang et al., 2018) proved that DNA replication was elevated in the LINC mutants larval muscles (by following both EdU incorporation, and FUCCI). Despite of this, no clear 2 fold increments in the DNA intensity in the mutant muscle nuclei was observed. At this stage we do not have a direct proof that DNA replication is performed along the entire genome. Nevertheless, the extent of elevated DAPI signal in the LINC mutants was comparable with that of BAF knock down nuclei.

3. E2F1. The authors observe a dependance of E2F1 enrichment at lamina on BAF. The authors assume that changes in this enrichment reflects function. The authors do not consider that the major part of E2F1 molecules are unaffected by BAF. Why do the authors believe that only the fraction of E2F1 at the nuclear envelope would be the functionally important fraction? The authors do not present any data supporting this hypothesis.

We show that E2F1 levels in the nucleoplasm are significantly elevated in BAF knockdown nuclei, and propose that this might explain the elevation in endoreplication observed (clarified in sentences in lines 257-259, 260-261, 328-330).

4. technical issues
The quantification would be more convincing, if an internal standard were used. I do not understand why quantifications were not normalised by the LaminC staining. An internal standard would avoid any mutant specific changes in morphology, such as imaging depth or nuclear morphology.
It is interesting to read that laminC behaves similar to BAF. This supports the idea that the LINC dependance of BAF is due to structural changes but not mechano-dependent. There are many more lamina proteins that can be employed as internal standards for proper quantifications such as Lamin Dm0, nuclear pore proteins, for example.

© 2020. Published by The Company of Biologists under the terms of the Creative Commons Attribution License (https://creativecommons.org/licenses/by/4.0/).

There is no point to normalize to lamin C or lamin Dm0 because their levels change in the mutants (as reported in Wang et al., 2015). In our quantifications we used Rolling Ball Background Subtraction package of Image J for background substraction. In addition in each of the experiments we incubated all larvae (mutant and control) in a single tube for fixation, and further labeling with antibodies mix. Imaging was performed at the same day under equal acquisition parameters. These ensure as much as possible equal conditions for experiments and their control. Notably, the measurements of BAF levels at the nuclear envelope were relative, e.g. the ratio between BAF at the nuclear envelope versus its levels at the cytoplasm, or nucleoplasm, so in this case there was no point for internal normalization.

Fig. 8C (previous version 6C) The distribution of nuclear E2F1/nuclear volume is bimodal. The symmetrical distribution in wild type splits into two population after baf depletion. one population has a lower intensity. the second population has a higher intensity. This indicates that there are two groups of the nuclei. It is not informative to put both populations together for such a bimodal distribution.
The presented bimodal distribution has nothing to do with the nucleolar localizaition.

Our accumulating data do not support the claim that the distribution of E2F1 is bimodal. I agree that there is higher variability between nuclei in the BAF knockdown, possibly due to variability in the efficiency of BAF knockdown.

expansion microscopy. I know that there has been a previous publication which introduced the term. Despite this the term expansion microscopy is misleading and questionable.

Since the original paper by Boyden group in 2015, cited 858 times the term "Expansion Microscopy" is routinely used and I do not find a reason to change it.

The quantification procedure included a context/region specific background subtraction (rolling ball). In my view it is mandatory in quantification to conduct any procedure on an image on all pixels to the same degree.
As similar results are obtained without the context specific image processing (rolling ball background subtraction), I am surprised that the authors still present data which are derived from a quantification procedure containing context specific image processing.

The method is used routinely in many publications and was recommended by the bioimaging experts in the Institute Imaging unit.

Reviewer 2 Advance summary and potential significance to field
This revised manuscript investigates the role of BAF during larval muscle development. It finds that BAF is localized at the nuclear membrane. This localization depends on the LINC complex, on intact sarcomere organization and the kinase Ball.
The authors find that reducing BAF, ball or the sarcomeric organization results in higher DNA content in the larvae, suggesting elevated endo-replication. Interestingly, the authors find a molecular link to a regulator of endo-replication, E2F, whose correct nuclear levels depend on Ball and BAF function.
The authors hypothesis that nuclear mechanics controls endo-replication, which needs to be regulated during larval growth.

As indicated in my initial review, I found these findings interesting, despite the missing characterization of the functional consequences after BAF knock-down.
Now, the authors added to the discussion that BAF knock-down larvae develop normally.

Reviewer 2 Comments for the author

1. The result that BAF knock-down larvae grow to adulthood is an important result, which should be document in the paper in the results part. Did the authors measure growth rate and developmental time?
Of course, knock-down levels can be one issue, however Baf knock-down is strong enough to cause the documented protein localization (E2F) and endo-replication defects.

© 2020. Published by The Company of Biologists under the terms of the Creative Commons Attribution License (https://creativecommons.org/licenses/by/4.0/).

Documentation of the experiment was added to the results. (217-223).

Further, the gain of function phenotype (BAF3D) could be explored.
If growth rate is normal in all cases, the functional link of the observed localization phenotypes in the BAF knock-down larvae, including the endo-replication defects, to larval growth is not established. This should then be clearly stated in the paper.

Description of the phenotype of larvae expressing the non phosphorytable form of BAF was added (lines 196-198).

2. The authors again introduce new data in the discussion as unpublished results. If these data are important for the paper, they should be added to the results section and shown. If they are not important, they should not be discussed as data not shown.

I removed the sentence regarding Ball localization in the LINC mutants from the Discussion described as data not shown (previously outlined in lines 308-312). Also the sentence describing the attempt to rescue koi mutants with GFP-BAF-3D (data not shown, described in previous manuscript in lines 308-313) was removed.

Reviewer 3 Advance summary and potential significance to field
This is a revised manuscript from Unnikannan et al. In previous work, the Volk lab has found an increase in DNA content in mutations in the LINC complex. In this work they provide data to support a linear pathway from the LINC complex to E2F1, a key regulator of DNA endoreplication. This pathway consists of localization of BAF via its phosphorylation status and Otefin at the inner nuclear envelope. This in turn regulates E2F1 localization at the nuclear envelope. Disruption of the Linc complex or its connection to the sarcomere, leads to release of BAF (and E2F1) from the nuclear envelope into the nucleoplasm where it leads to DNA endoreplication.

Reviewer 3 Comments for the author
In this revised version, the authors present a clearer set of data and a streamlined message, in which they implicate the LINC complex in the localization of BAF at the nuclear envelope which in turn localizes, in some way, E2F1. E2F1 in turn regulates endoreplication. This mechanism explains the change in DNA content shown in the LINC mutations.
However there remain some concerns with the data as presented:

1.Based on published literature in other systems, the authors assume that, in the Drosophila larval muscle system, that the LINC complex functions as a mechanotransducer. They interpret altered nuclear shape in the LINC mutants as “altered mechanotranduction”. However, the authors offer no direct measure of this. There can be other explanations for the altered shapes which have not been ruled out. Hence the “nuclear mechanics” connection in the title and in the results should be removed. The authors are free to speculate about mechanics in the Discussion section.

The title was changed as well as the relevant sentences in the results (marked with blue).

2.Similarly with the Otefin data, the authors assume that, since BAF has been shown in other systems to bind to Otefin, it would do the same here. No data has been shown to support this. In addition to a co-ip, the authors could show a change in Baf nuclear envelope localization in an Otefin mutant/knockdown. And what about the other LEM domain proteins? Why are they ruled out? In Otefin mutants, is there increased DNA content? Is E2F similarly mislocalized as would be predicted? The model is not fully tested.

Throughout the text we emphasized that our model is based on our data, as well as on data from other organisms. This is now better delineated, for example see line 144 (added in blue “as described in other organisms”).

Other issues:
The GFP-BAF, GFP-BAF-3A, GFP-BAF3D – all the levels are different in the images.
Could this influence the expression localization that is reported for each? Do E2F1 localization and DNA content change in these backgrounds as a test of the authors’ model?

© 2020. Published by The Company of Biologists under the terms of the Creative Commons Attribution License (https://creativecommons.org/licenses/by/4.0/).

Western blot analysis indicated that although we used staged larvae and a single mef2-GAL4 driver the levels of GFP-BAF, GFP-BAF3D, and GFP-BAF3A in the muscle nuclei differed significantly, possibly due to differential stability of the GFP-BAF proteins. This variability is reflected by the representative images in Fig 6. Furthermore, we noted that overexpression of the BAF-GFP constructs had additional effects on the muscles which could indirectly affect the DNA content and E2F1 localization. For example, overexpression of BAF-GFP often led to disruption of the nuclear envelope and leakiness of DNA from the nucleus to the cytoplasm. Overexpression of BAF-GFP-3A led to disruption of the muscle tissue and often larval death. We think that these differential dominant outcomes do not allow objective comparison between E2F1 localization and DNA content in the muscles of each of the BAF protein species.

BAF total levels are reduced in the SLS RNAi, could that contribute to reduced BAF localization at the nuclear envelope?

BAF levels indeed reduced in the sls RNAi, however we show in Figure 4H and 4I that the relative levels of BAF at the nuclear envelope versus the cytoplasm (H) or nucleoplasm (I) decreased, indicating that despite total BAF reduction in the muscle fiber its relative levels at the nuclear envelope are specifically reduced (see lines 160-163).

Baf reduction appears to have a greater impact on the DNA content than Ball KD or sls KD. How does this compare to LINC kd? How are E2F1 levels altered in these situations as a further test of the model?

It is difficult to compare between the relative reduction of DNA in baf KD, sls KD and ball KD because the experiments were performed separately. This is the reason that each experiment is shown alone (e.g. Fig 7, A, B, C).

The authors should show the remaining Baf localization in the BAF kd .. are all the levels reduced or more so at the nuclear envelope?

As shown in Supplemental Fig S1 the levels of BAF are significantly reduced in all subcellular localizations. I added a sentence to clarify this further (217-219).

The authors should also
clarify their comment in the discussion line 298-300 “These findings are consistent with a dynamic exchange of BAF between cytoplasmic and nuclear pools, where BAF in the cytoplasm primarily responds to mechanical signals” . This appears at odds with what the authors are proposing in their model.

We revised the sentence to clarify its meaning (lines 306-311).

<u>Third decision letter</u>

MS ID#: DEVELOP/2020/191304

MS TITLE: Recruitment of BAF to the nuclear envelope couples between LINC complex function and endoreplication

AUTHORS: C.P. Unnikannan, Adriana Reuveny, Dvorah Grunberg, and Talila Volk

I have now received all the referees reports on the above manuscript, and have reached a decision. The referees' comments are appended below, or you can access them online: please go to BenchPress and click on the 'Manuscripts with Decisions' queue in the Author Area.

© 2020. Published by The Company of Biologists under the terms of the Creative Commons Attribution License (https://creativecommons.org/licenses/by/4.0/).

The overall evaluation is positive although one of the reviewers is not satistifed with a few points. I looked at the manuscript and in lughht of this and considering all the reviewers comments, I am happy to consider publication in Development though the title should be changed as proposed by Rev 3.

Reviewer 1

Advance summary and potential significance to field

..

Comments for the author

..

Reviewer 2

Advance summary and potential significance to field

It is a bit disappointing to read in the second revision of this manuscript for the first time that 'the full physiological contribution of BAF to muscle growth awaits experiments in which a more efficient reduction of BAF levels in the muscle tissue will be induced' , as we now have just learnt that BAF RNAi larvae develop normally.

Comments for the author

Other than that I have no more comments, as all my other questions have been answered.

Reviewer 3

Advance summary and potential significance to field

The authors present experiments that support that the LINC complex, through its Otefin, regulates the interaction of BAF, which, in turn, controls the localization of E2F1, a key regulator of endoreplication. The linkage between these different protein complexes builds and extends previous work in the field.

Comments for the author

The authors have been responsive to the last review and have clarified issues raised.

Please note that the title should be revised as the title structure needs improvement. Consider "Recruitment of BAF to the nuclear envelope couples the LINC complex to endoreplication"

section title: line 149-150
Title is unclear: "nuclear sarcomeres"?
Perhaps BAF localization at the nuclear envelope depends on association of nuclei with nearby sarcomeres.

Third revision

Author response to reviewers' comments

In response to Reviewer no 3 I have changed the title, and the section title in line 149-150.

© 2020. Published by The Company of Biologists under the terms of the Creative Commons Attribution License (https://creativecommons.org/licenses/by/4.0/).

Fourth decision letter

MS ID#: DEVELOP/2020/191304

MS TITLE: Recruitment of BAF to the nuclear envelope couples the LINC complex to endoreplication

AUTHORS: C.P. Unnikannan, Adriana Reuveny, Dvorah Grunberg, and Talila Volk
ARTICLE TYPE: Research Article

I am happy to tell you that your manuscript has been accepted for publication in Development, pending our standard ethics checks.

© 2020. Published by The Company of Biologists under the terms of the Creative Commons Attribution License (https://creativecommons.org/licenses/by/4.0/).
